# Systematic identification and functional characterization of the CFEM proteins in fishscale bamboo rhombic-spot pathogen *Neostagonosporella sichuanensis*

**DOI:** 10.3389/fpls.2024.1396273

**Published:** 2024-05-31

**Authors:** Fang Liang, Lijuan Liu, Chengsong Li, Yinggao Liu, Shan Han, Hua Yang, Shujiang Li, Wenkai Hui, Long Liu, Chunlin Yang

**Affiliations:** ^1^ College of Forestry, Sichuan Agricultural University, Chengdu, China; ^2^ National Forestry and Grassland Administration Key Laboratory of Forest Resources Conservation and Ecological Safety on the Upper Reaches of the Yangtze River and Forestry Ecological Engineering in the Upper Reaches of the Yangtze River Key Laboratory of Sichuan Province, College of Forestry, Sichuan Agricultural University, Chengdu, China

**Keywords:** *Neostagonosporella sichuanensis*, effector, CFEM domain, plant immunity, stress tolerance, virulence

## Abstract

Fungal effectors play a crucial role in the interaction between pathogenic fungi and their hosts. These interactions directly influence the invasion and spread of pathogens, and the development of diseases. Common in fungal extracellular membrane (CFEM) effectors are closely associated with the pathogenicity, cell wall stability, and pathogenic processes of pathogenic fungi. The aim of this study was to investigate the role of CFEM proteins in *Neostagonosporella sichuanensis* in pathogen-host interactions. We retrieved 19 proteins containing CFEM structural domains from the genome of *N. sichuanensis*. By systematic analysis, five NsCFEM proteins had signal peptides but lacked transmembrane structural domains, and thus were considered as potential effectors. Among them, NsCFEM1 and NsCFEM2 were successfully cloned and their functions were further investigated. The validation results show that NsCFEM1 was localized in the cell membrane and nucleus, whereas NsCFEM2 was exclusively observed in the cell membrane. Both were identified as secreted proteins. Additionally, NsCFEM1 inhibited Bax-induced programmed cell death in *Nicotiana benthamiana*, whereas NsCFEM2 did not induce or inhibit this response. NsCFEM1 was implicated as a virulence factor that contributes to fungal growth, development, stress response, and pathogenicity. NsCFEM2 was implicated in maintenance of cell wall stability. This study lays a foundation for elucidating the role of CFEM proteins in the pathogen of fishscale bamboo rhombic-spot caused by *N. sichuanensis*. In particular, the functional studies of NsCFEM1 and NsCFEM2 revealed their potential roles in the interaction between *N. sichuanensis* and the host *Phyllostachys heteroclada*.

## 1 Introduction

*Phyllostachys heteroclada* Oliver is prevalent in southwest China and is a key crop for edible shoots and bamboo timber, and a vital food source for giant pandas in the wild (Shi and Chen, 2022). *Neostagonosporella sichuanensis* is the predominant pathogenic agent responsible for rhombic-spot diseases in *P. heteroclada* forests (Yang et al., 2019). This affliction primarily targets the culms, branches, and exposed rhizomes of mature bamboo > 0.5 years of age. The affected areas exhibit distinctive rhombic spots, leading to the development of withered plants, branch withering, and tip withering in diseased bamboo. In severe cases, this pathology has significant adverse effects on the yield and quality of bamboo (Qi et al., 2021). Rhombic-spot of *P. heteroclada* is a strongly pathogenic disease that is extremely harmful to bamboo culms (Zheng et al., 2022).

In the dynamic interplay between pathogens and host plants, pathogens utilize diverse mechanisms to overcome defense systems of plants, gain access to the plants for nutrient absorption, or disrupt the regular growth patterns of the plants (Jones and Dangl, 2006). Pathogens excrete substantial quantities of various proteins and compounds to obstruct or disrupt the host plants defensive responses (Hogenhout et al., 2009). Therefore, knowledge of the interaction between plants and pathogenic fungi is crucial for the understanding of the mechanics of pathogenicity of these pathogens (Cesari, 2018; Han, 2019). Pathogen effectors inhibit either PAMP-triggered immunity (PTI) or effector-triggered immunity (ETI). The intensity of response differs between PTI and ETI, with ETI leading to a strong hypersensitive response (HR), which occurs at the site of pathogen infestation and prevents further spread of the pathogen (Tsuda and Katagiri, 2010; Wang et al., 2019; Yuan et al., 2020). Recent evidence indicates a growing understanding that PTI and ETI collaborate with a significant overlap in signalling pathways and their downstream responses (Ngou et al., 2020; Yuan et al., 2020). Effector proteins are pivotal in the infection process of pathogenic fungi or oomycetes by entering host cells through specific secretion systems and transfer mechanisms, subsequently executing their functions within the host cytoplasm, nucleus, or extracytoplasmic space (Kulkarni et al., 2003). Most of the effector protein families secreted by pathogen have been efficiently screened and successfully identified (Dou and Zhou, 2012; Yuan et al., 2020; Chang et al., 2022). Most pathogenic fungi secreted effector proteins harbor a conserved motif at their N- or C-terminal extremities, like RxLR, CRN, CFEM (common in fungal extracellular membrane), LYSM, RGD, DELD, EAR, RYWT, and Y/F/WXC (Wang et al., 2022). Exploring the function of effectors, particularly those with conserved motifs, is important for understanding symbiotic or pathogenic mechanisms between hosts and pathogens, developing defense strategies, and providing insights into variety selection, crop improvement, and resistance to abiotic stresses (Kulkarni et al., 2003).

The CFEM domain is exclusive to fungi and features a structural domain with a conserved motif, PxC[A/G]x_2_Cx_8–12_Cx_1–3_[x/T]Dx_2–5_CxCx_9–14_Cx_3–4_Cx_15–16_C (x represents any residue, with its specific range indicated). The protein’s structural domain consists of approximately 60 amino acids (Appella et al., 1988; Zhang et al., 2015). The CFEM structural domain-containing proteins were initially defined as glycosylphosphatidylinositol-anchored (GPI)-anchored cell wall proteins that serve as extracellular sensors (Zhang et al., 2015). The CFEM structural domain incorporates eight spacer-distributed semideaminic acid residues that are analogous to the structural domains of epidermal growth factors, which function as extracellular receptors and signal transducers (Lamarre et al., 2000; Zhang et al., 2015). Proteins containing the CFEM structural domain have been found in various fungal pathogens, for instance *Magnaporthe oryzae*, which contains approximately 61 CFEM proteins (Ramanujam et al., 2013); *Colletotrichum graminicola*, which contains approximately 32 CFEM effectors (Zhang et al., 2017); and *C. gloeosporioide*, which contains approximately 22 CFEM effectors (Chen et al., 2013). Certain effector proteins, for instance the Pth11 effector protein found *Magnaporthe oryzae* that facilitates appressorium formation, have demonstrated pivotal roles in pathogenicity (Kou et al., 2017). Other examples include the Csa2 effector from *Candida albicans*, which utilizes hemoglobin as a source of iron (Okamoto-Shibayama et al., 2014), the BcCFEM1 effector in *Botrytis cinerea* exhibits pivotal roles in pathogenicity, spore production, and external stress tolerance (Zhu et al., 2017), CFEM A-C effector in *Aspergillus fumigatus*, which contributes to the stabilization of the cell wall (Vaknin et al., 2014). Analogously, CgCcw14 and CgMam3 in *Candida glabrata* are instrumental in maintaining intracellular iron content, adherence to epithelial cells, and virulence (Srivastava et al., 2014). However, CFEM-containing proteins in *N. sichuanensis* have yet to be systematically identified and functionally analyzed.

In this study, bioinformatic analysis was used to predict 19 CFEM proteins from the *N. sichuanensis* genome. Five NsCFEM candidate effector proteins have been identified based on the criteria for defining effector proteins. Of the five, two were successfully amplified. The data represent the first identification of CFEM effectors in *N. sichuanensis*, providing a foundation for exploring their functions and enhancing our comprehension of the interaction mechanisms between *N. sichuanensis* and its host *P. heteroclada* at the molecular level.

## 2 Materials and methods

### 2.1 Plants, strains, and culture conditions

Healthy fish-scale bamboo seedlings were transplanted from Ya’an to the Chengdu Academy of Agriculture and Forestry Sciences in Chengdu City, Sichuan Province, China. *Nicotiana benthamiana* was cultivated in an artificial climate incubator at 25°C with a 16h light period. *N. sichuanensis* wild type strain (SICAUCC 16–0001) was obtained from diseased fish-scale bamboo in Ya’an City, Sichuan Province, China, inoculated on PDA agar medium and then incubated at 25°C for 30 d. *Escherichia coli* DH5α competent cell and *Agrobacterium tumefaciens* GV3101 (pSoup-19) competent cell were individually cultured on Luria-Bertani agar media. The positive DH5α colonies were incubated in liquid LB media under oscillation at 200 rpm and 37°C, while the GV3101 colonies were cultured similarly at 28°C. Additionally, the *Saccharomyces cerevisiae* YTK12 strain was cultured on yeast extract peptone dextrose agar media in the dark at 30°C. The PGR107 vector, PCAMBIAsuper1300-GFP, and *N. benthamiana* plants were donated by the Forest Pathology Laboratory of Sichuan Agricultural University. The yeast strain YTK12 and the pSuc2 vectors were provided by Professor Shuangcheng Li from the Rice Research Institute, Sichuan Agricultural University, China).

### 2.2 Bioinformatic identification of CFEM-containing proteins in *N. sichuanensis*

The initial CFEM protein to be discovered is MoACI1, a protein that interacts with adenylate cyclase (MAC1) and was identified in the *M. oryzae* (Kulkarni et al., 2003). To screen for the CFEM effector in *N. sichuanensis*, we utilized the ACI1, a CFEM domain-containing protein from *M. oryzae* as a reference (Gong et al., 2020). Utilizing the BLASTP algorithm with an e-value threshold set at < 1e-10, we searched the *N. sichuanensis* genome database (whole-genome data not publicly available). Subsequently, the protein sequences in the whole genome of *N. sichuanensis* were carefully searched for the presence of the CFEM domain through Pfam on the SMART website (http://smart.embl-heidelberg.de/). Only the sequences harboring CFEM domains were considered for further analysis. For the analysis of signal peptide sequences in the NsCFEM proteins, we leveraged the SignalP 4.1 Server (http://www.cbs.dtu.dk/services/SignalP/). Additionally, we predicted the TM domain of the NsCFEM proteins using the TMHMM tool (http://www.cbs.dtu.dk/services/TMHMM/). Finally, the subcellular localization of the NsCFEM proteins was forecasted utilizing the TargetP 2.0 Server (https://services.healthtech.dtu.dk/service.php?TargetP-2.0).

### 2.3 Phylogenetic analysis and multiple sequences alignment

A comprehensive phylogenetic analysis was performed to construct a maximum likelihood tree encompassing 19 NsCFEM proteins using the MEGA 7.1 software. Bootstrap evaluation employing the P-distance model with 1000 replications. To gain further insight, we analyzed the CFEM structural domains, signal peptides, and transmembrane domains of 19 NsCFEM proteins sequences. To investigate the conserved amino acid patterns of the structural domains in NsCFEM in more depth, we extracted the CFEM structural domain sequences and aligned the sequences for multiple sequence comparison using ClustalW software (Han et al., 2016). For this purpose, we utilized two reported CFEM structural domains of *Candida albicans* (Csa2, XP_713316.1) and *M. oryzae* (ACI1, AAN64312.1) as reference templates. The results were modified and visualized using ESPript 3.0 (https://espript.ibcp.fr/ESPript/cgi-bin/ESPript.cgi) and WebLogo (http://weblogo.berkeley.edu/logo.cgi) comparisons, and provide the detailed and visual representation of the conserved amino acid motifs in NsCFEM proteins.

### 2.4 Protein model analysis of candidate NsCFEM effectors with Phyre2 Server

Candidate effectors were identified as proteins containing N-terminal signal peptides but lacking transmembrane regions. The models of five NsCFEM effectors were protein 3D model utilized the Phyre2 website, a web tool specialized in predicting and analyzing protein models, runnable at http://www.sbg.bio.ic.ac.uk/phyre2 (Nasser et al., 2016). Crystal Structure of the CFEM protein Csa2 in *C. albicans* with adenine retrieved from the PDB database (PDBID: 4y7s). The CFEM domain sequences of the five NsCFEM effectors were uploaded to the Phyre2 website and aligned accordingly. PSI-Blast was employed to search for homologous sequences, and a Hidden Markov Model structure was constructed based on these homologies.

### 2.5 Comparative analysis of model quality assessment and structural overlays

Prediction of tertiary structures of five candidate NsCFEM proteins using protein tertiary structure prediction software Alphafold2 (Yang et al., 2023). To ensure the high quality of the predicted protein models and the validity of the subsequent analysis results, two quality assessment software, PROCHECK (Hodsdon et al., 1996), and Qmean (Benkert et al., 2009), were used to assess the quality of the predicted models. The 3D model of Csa2 was superimposed and compared with 3D models of the five NsCFEM effector proteins by means of the UCSF Chimera software (Pettersen et al., 2004).

### 2.6 Hypersensitive response assay and subcellular localization

Hypersensitive response assays and subcellular localization of NsCFEM effectors were conducted using the agroinfiltration method (Gong et al., 2020). Specific primers were designed using Premier software (version 5.0) and the CDS regions of NsCFEM1 and NsCFEM2 with homology arms were amplified by PCR. Homologous recombination was performed at the *Bam*HI site of the PGR107 vector and the *Bam*HI site of the PCAMBIAsuper1300-GFP vector. Bax is a death-promoting member of the Bcl-2 protein family that induces programmed cell death (Lacomme and Santa Cruz, 1999). Bax served as a positive control in an infiltration assay on *N. benthamiana*. Finally, the two recombinant vectors obtained (pGR107-NsCFEM and GFP-NsCFEM) were transformed into *A. rhizogenes* GV3101 competent cells, respectively, and coated on LB agar medium and incubated at 28°C for 48 h. Positive colonies were picked to expand the culture, and the precipitates were collected by centrifugation and treated with injection The precipitate was washed three times with injection buffer. The injection buffer was formulated as 10 mmol/L MgCl_2_, 10 µmol/L acetosyringone (AS) and 10 mmol/L 2-(N-morpholino)ethanesulfonic acid (MES), and the mixture was adjusted to a pH of 5.6.) After resuspension, an appropriate amount of injection buffer was added to an OD600 of 0.6–0.8 and left to stand at 28°C for 2h, protected from light, and injected into 4- to 5-week-old *N. benthamiana* leaves using a syringe for removing needles (Sparkes et al., 2006). The infected tobacco plants were inspected after 2 days of incubation and observed by confocal laser microscopy using a model FV3000 microscope (Olympus, Tokyo, Japan) as a localization test. Furthermore, symptoms of cell death in *N. benthamiana* leaves were observed and recorded within 4–5 d after inoculation.

### 2.7 Yeast secretion trap screen assay

We employed a yeast signal sequence capture assay to further verify whether the signal peptides of the NsCFEM1 and NsCFEM2 effector proteins have a secretory function. The predicted signal peptide sequences of NsCFEM1 and NsCFEM2 effector proteins were amplified by PCR using specific primers, and the signal peptide sequences were homologously recombined into the two restriction sites, *Eco*RI and *Xho*I, of the pSUC2 vector (Jacobs et al., 1997; Yin et al., 2018). The selected pSUC2 vector contains the tryptophan synthesis gene, the sucrose convertase gene (SUC2) and a deletion signal peptide. Ligating the signal peptide sequences of the NsCFEM1 and NsCFEM2 effector protein to the SUC2 sucrose convertase gene before. When a functional signal peptide is attached to pSUC2, the secretory function of sucrose convertase can be restored. The transformed yeast was evenly spread on CMD-W agar medium at 30°C for 48 h. If the yeast grows on the CMD-W agar medium, the positive colonies can be transferred to YPRAA agar medium. If the positive colony can continue to grow on YPRAA agar medium, it can be proved that the signal peptide sequence of the NsCFEM protein has a secretion function. On the other hand, if the positive colony cannot continue to grow on YPRAA agar medium, the signal peptide sequence of the NsCFEM protein has no secretion function. If the signal peptide of the NsCFEM protein is predicted to have a secretory function, sucrose convertase can be secreted into extracellular action to reduce 2,3,5-triphenyltetrazolium chloride (TTC) to the red precipitate 1,3,5-triphenylstyrene (TPF). This method can be utilized to further determine whether the signal peptides of NsCFEM1 and NsCFEM2 have secretory functions (Yin et al., 2018). The positive colonies were inoculated into 10 mL of YPDA liquid medium, incubated at 30°C, 220 r/min for 2 d, centrifuged at 12000 r/min for 1 min, collected and washed twice with ddH_2_O, and finally resuspended in 750 μL of sterile water. Then add 250 μL of 10 mmol/L acetic acid-sodium acetate buffer (pH=4.7) and 500 μL of 10% sucrose solution, and incubate for 10 min at 37°C, then centrifuge for 1 min at 12000 r/min. Take out 100 μL of supernatant, add 900 μL of 0.1% TTC, and let it stand for 5 min to observe the color change. If the color changes (colorless to red) and a precipitate is produced, the signal peptide of the NsCFEM protein has a secretory function.

### 2.8 Construction of gene knockout and back-construction vectors

Hygromycin B and G418 (Lee et al., 2010; Chen et al., 2016) used in the *N. sichuanensis* genetic transformation screen were selected as antibiotic markers for screening the knockout and complement transformants, respectively. Taking the CDS region of NsCFEM1 and NsCFEM2 as the central point, we selected 1500 bp both upstream and downstream as the homologous arms. These regions were utilized to design primers specifically aimed at amplifying the homologous arms of NsCFEM1 and NsCFEM2 from the DNA of *N. sichuanensis*. The hygromycin transferase gene (*hph*) fragment containing the promoter was amplified using the pCAMBIA1300ura vector. Primer design software CE Design (http://www.vazyme.com) was used to design upstream and downstream homology arms and hph fragments with complementary sequences. The knockout vector (NsCFEM1–5’ + hph+ NsCFEM1–3’ and NsCFEM2–5’ + hph+ NsCFEM2–3’) was constructed by reacting the three fragments with linkers and the pCE-Zero linearized vector after single digestion with *Eco*RI through 2× ClonExpress Mix for multi-fragment homologous recombination. The principle of constructing knockout vectors is shown in **Supplementary Figure 1**.

Genetic complementation validation guides the complete gene back to the gene-blocking knockout strain. This excludes polar effects or spontaneous mutations to ensure that the effect occurs after the gene is blocked. In this study, genomic DNA of the wild type *N. sichuanensis* was used as a substrate to amplify the target gene and its required promoter complementary sequence. The general promoter was within 1500 bp upstream of the start codon. This complementary sequence was cloned into the *Kpn*I site of the pEASY-NeoR vector containing G418 to obtain complementary expression vectors (Promoter-NsCFEM1 and Promoter-NsCFEM2).

### 2.9 Protoplast preparation and genetic transformation

*N. sichuanensis* protoplasts were prepared using lysine and driselase (Sigma-Aldrich, St. Louis, MO, USAIGMA) as previously described (Goswami, 2012). The strain was activated in advance, and fresh mycelium from single colonies was transferred into M3S liquid medium, and cultivated under shaking conditions at 25°C and 130 rpm for a duration of 3 to 4 d. A solution of Driselease (Sigma-Aldrich) was configured in a 50 mL centrifuge tube. Enzyme catalyzed lysis occurred during stirring at 4°C for 20 min. The sample was centrifuged at 3500 rpm and 4°C for 10 min. The supernatant containing mycelia was filtered through a monolayer of Miacloth, and washed 3–5 times with 20 mL of 1.2 mol/L KCl solution. A suitable amount of mycelium was picked and placed in another solution of the enzyme and incubated at 30°C and 60 rpm for 4 h. The mycelium after enzymatic digestion was filtered using a Miacloth bilayer, washed several times with 20 mL of 1.2 mol/L KCl solution, and centrifuged at 3000 rpm and 4°C for 10 min. The supernatant was discarded. The white precipitate on the tube wall was protoplasts. Fifteen milliliters of 1.2 mol/L KCl solution was added to rinse and mix the protoplasts before centrifugation. The supernatant was discarded, and 1 mL of STC buffer was added and the protoplasts were quickly resuspended to avoid rupture. The protoplasts were observed by BX43 biological microscope (Olympus in the Japanese), counted on a hemocytometer, and their concentration was adjusted to 1×10^7^ to 1×10^8^ cells/mL using STC buffer.

A slight modification of previous methods using polyethene glycol (PEG)-mediated transformations (Fang et al., 2021). A 200 μL volume of the protoplast preparation was added to a 50 mL sterile centrifuge tube, followed by the addition of 5–10 ng fusion knockout DNA fragment and incubation in an using an ice bath for 20 min. A total of 1200 μL of 40% PEG was added along the wall of the tube three times and mixed. The resulting suspension was incubated in an ice bath for 20 min. Ten microliters of TB3 liquid medium was added, mixed well, and tilted in a constant temperature shaking incubator for 25°C at 90 rpm for 9 h. The resuscitation of protoplasts was then examined by microscope. After successful resuscitation of the knockout transformants, 40 mL of TB3 Bottom Agar (including final concentrations of 50 μg/mL of Hyg and 50 μg/mL of Amp) was added and cultured for 10 h. TB3 top agar was again coated using the same method, except for a higher final concentration of Hyg (150 μg/mL) at 25°C for 10 days. The final concentration of G418 used in the complementary strains screening process was 50 μg/mL of TB3 bottom agar and 250 μg/mL of TB3 top agar. Single transformants were selected for inoculation onto PDA plates containing high concentrations of resistance markers, selected by three passages, and verified by PCR. The principle of the PCR method to validate the transformants is shown in **Supplementary Figure 1**.

### 2.10 Phenotypic and stress treatment analysis of knockout and complementary strains

Nutritional growth differences between wild type, knockout and complemented strains were analyzed. The wild-type, knockout and complementation strains were inoculated on PDA plates and cultured at 25°C for 30d. Every 5 days, the mycelium diameter was measured by crossover method and growth was observed. Three replicates were set up for each strain and the whole experiment was repeated three times. Qi et al. (2021) described that *N. sichuanensis* can grow indoors, but no sporulation was observed. We observed that the liquid extracts of bamboo poles and lactose-containing agar medium induced sporulation. Therefore, each strain was inoculated separately on lactose agar medium with decoction of bamboo poles and cultured at 25°C for 30d. Mycelium was rinsed with 4 mL of deionized distilled water and spores were collected, spore morphological characteristics were observed under a microscope, and spore production was determined by hemocyte counting plate counting method. The collected spores were diluted to a concentration of 1×10^4^ conidia/mL, and then 20 μL of spore suspension was added dropwise onto glass slides, and the spores of each strain were incubated separately for 24 hours in the dark at 25°C. Germination, formation of normal colonies, and reproduction were observed by microscope. Three replicates were set up for each strain and the whole experiment was repeated three times. To assess the differences in stress response among the strains, a stress sensitivity assay was performed using PDA supplemented with different agents: 1 M NaCl, 1 M sorbitol, 0.4 mg/mL Calcofluor White, 20 mM hydrogen peroxide, 0.8 mg/ml Congo Red, and 0.02% sodium dodecyl sulfate at 25°C for 30 d (Zhu et al., 2017). All experiments were replicated thrice, and the resulting data were subjected to statistical analysis using one-way ANOVA and Duncan’s range test, implemented in SPSS 16.0 software (IBM, Armonk, NY, USA), to determine significant differences between pairs of means.

### 2.11 Pathogenicity test of knockout and complementary strains

Five one-year-old healthy fishscale bamboo were randomly selected for determination of pathogenicity in each experimental group. Before inoculation, the surface of the bamboo culm was cleaned with sterile water, branches were gently wiped with a skimmed cotton ball soaked with 70% alcohol, and the surface of the bamboo culm was destroyed using a scald. Wild type pathogen discs (9 mm diameter) from PDA were added to 150 mL of PDB medium containing glass beads, incubated in a mycelium ball attached to the 1-year bamboo injury site of the pot, and wrapped to the inoculation site with sterile gauze. The remaining 25 fishscale bamboo were inoculated with a mycelium ball of ΔNsCFEM1 and ΔNsCFEM2 knockout strains, mycelium ball of complementary strain ΔNsCFEM1+ and ΔNsCFEM2+, and blank agar blocks without mycelium, with five fishscale bamboo per treatment. The samples were bagged in a moist environment. Fresh mycelium balls were used to replace existing balls every 2 days. The symptoms of the plants were observed using conventional cultivation management methods. Disease occurrence was investigated after 45 days, and the diameter of damage of each plant was recorded.

## 3 Results

### 3.1 Bioinformatics identification of CFEM proteins in *N. sichuanensis*

Using the ACI1 protein of *M. oryzae* as a reference, nineteen CFEM proteins (NsCFEM1–19) were retrieved from the genome of *N. sichuanensis*, using BLASTP analysis (Kulkarni et al., 2003). The lengths of these proteins ranged from 174 amino acids (aa) for NsCFEM13 to 1652 aa for NsCFEM16). Further confirmation of the CFEM domains was carried out through SMART analysis, revealing that all CFEM domains, except for those in NsCFEM1, 10, and 13, were situated were located near the N-terminus of the respective proteins. Subsequently, signal peptides were predicted using the SignalP Server, indicating that only 11 NsCFEMs possessed a predicted signal peptide at their N-termini. Among these 11 proteins, six NsCFEMs (NsCFEMs 5, 7, 8, 13, 16, and 18) contained transmembrane structural domains. The protein identification numbers and analyses results are listed in **Table 1**.

**Table 1:** The identification of CFEM domain protein in *Neostagonosporella sichuanensis*.

Name	Protein ID	Amino acid (aa)	Position of CFEM domain (aa)	TM no^1)^.	SP cleavage^2)^	mTP^3)^	SP	Other^4)^	Loc^5)^	RC^6)^
NsCFEM1	PP279572	636	399–464	0	18/19	0.050	0.796	0.221	S^5^)	3
NsCFEM2	PP279573	219	56–121	0	21/22	0.361	0.845	0.009	S	3
NsCFEM3	PP279574	278	98–163	0	-^2^ **^)^**	0.639	0.049	0.350	S	4
NsCFEM4	PP279575	511	37–93	0	20/21	0.043	0.910	0.058	S	1
NsCFEM5	PP279576	402	47–108	1	30/31	0.768	0.098	0.099	S	2
NsCFEM6	PP279577	1043	14–81	0	15/16	0.201	0.067	0.726	^-5^)	3
NsCFEM7	PP279578	429	24–87	7	19/20	0.085	0.846	0.122	S	2
NsCFEM8	PP279579	551	32–96	7	23/24	0.103	0.894	0.018	S	2
NsCFEM9	PP279580	445	6–53	6	-	0.040	0.328	0.909	-	3
NsCFEM10	PP279581	388	200–265	0	-	0.467	0.046	0.571	-	5
NsCFEM11	PP279582	238	20–82	0	18/19	0.073	0.713	0.09	S	2
NsCFEM12	PP279583	967	40–96	7	-	0.244	0.045	0.767	-	3
NsCFEM13	PP279584	174	101–165	1	18/19	0.579	0.2	0.078	S	4
NsCFEM14	PP279585	523	67–127	6	-	0.165	0.116	0.764	-	3
NsCFEM15	PP279586	517	112–175	7	-	0.569	0.064	0.378	S	5
NsCFEM16	PP279587	1652	399–464	8	18/19	0.05	0.796	0.211	S	3
NsCFEM17	PP279588	561	138–201	8	-	0.525	0.105	0.29	S	4
NsCFEM18	PP279589	705	28–80	5	19/20	0.028	0.978	0.025	S	1
NsCFEM19	PP279590	393	107–170	1	-	0.554	0.1	0.209	S	4

1) TM, transmembrane domain.

2) SP cleavage, cleavage site of signal peptide (SP) in the NsCFEM proteins; –, no prediction.

3) The mTP, mitochondrion localization.

4) Other, any other localization.

5) Loc: Prediction of localization (S is secretion pathway; –, no prediction).

6) RC: Probabilistic estimated values of subcellular localization, the confidence is much higher with the value closer to 1.

### 3.2 Bioinformatics analysis of CFEM domain

**Figure 1:**
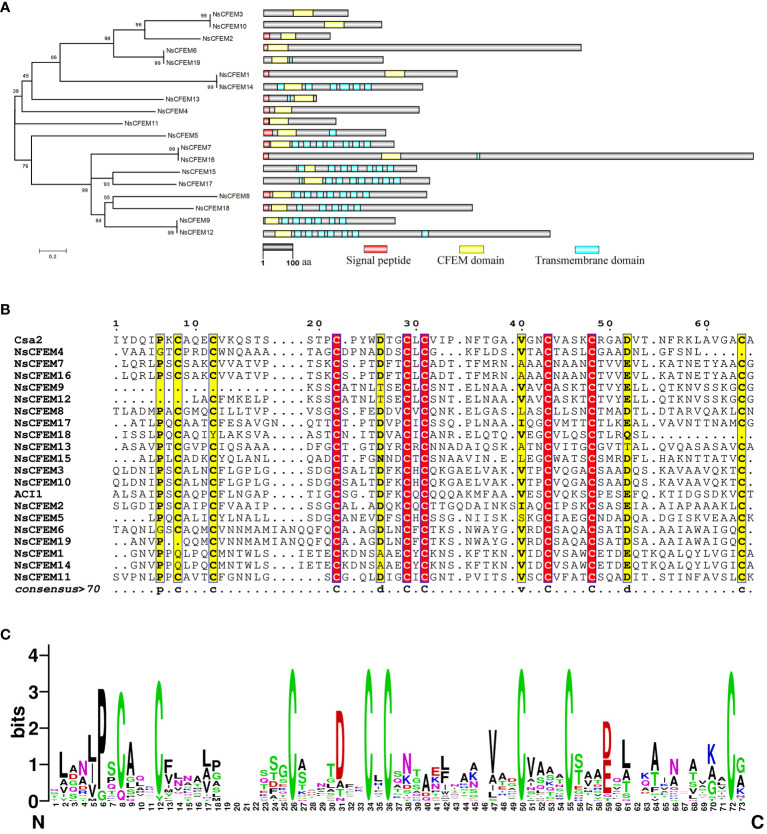
Bioinformatics analysis of CFEM domain proteins in *N. sichuanensis*. **(A)** Phylogenetic analysis of CFEM domain proteins in *N. sichuanensis*. Neighbor-joining phylogenetic tree based on amino acid sequences of 19 NsCFEM proteins was created. The numbers shown on the nodes indicate the percentage of 1000 bootstrap replicates in which they occur. On the right is a structural map of the NsCFEM proteins, with the transmembrane structural domains indicated by blue boxes, the CFEM structural domains indicated by yellow boxes, and the signal peptides indicated by red boxes. The scale bar indicates 100 amino acids. **(B)** Amino acid sequence comparison of the CFEM domains of 19 NsCFEM proteins. Amino acid sequences within the CFEM domains of 19 NsCFEM proteins were aligned using ClustalW, and the results were modified using ESPript 3.0. **(C)** The WebLogo visualization depicts the CFEM domains from 19 NsCFEM proteins. Conserved amino acids are highlighted in descending order of conservatism, with green, black, and red representing the highest to the lowest levels of conservation. The size of each amino acid symbol represents the degree of conservatism, determined through the WebLogo algorithm, providing a visual representation of the amino acid conservation patterns within the CFEM domains.

Based on the amino acid sequences of the 19 NsCFEM proteins, we constructed a neighbor-joining phylogenetic tree (**Figure 1A**). The analysis showed that these NsCFEM proteins could be broadly categorized into two groups: the first group contained eight NsCFEM proteins (NsCFEM7, 8, 9, 12, 15, 16, 17, and 18), which had multiple transmembrane regions; the second group consisted of 11 NsCFEM proteins (NsCFEM1, 2, 3, 4, 5, 6, 10, 11, 13, 14, and 19), which have few or no transmembrane regions. The effector proteins secreted by fungi play a key role in intracellular protein translocation and are usually guided by signaling peptides. All NsCFEM proteins except NsCFEM3, 9, 10, 12, 14, 15, 17, and 19 possess an N-terminal signal peptide and are therefore predicted to be secreted proteins.

To gain further insights into the conserved amino acids (aa) within the CFEM domains, multiple sequence alignments were conducted (**Figure 1B**), utilizing two known proteins that contain CFEM domains as references: Csa2 from *C. albicans* and ACI1 from *M. oryzae*. All of the chosen NsCFEM proteins shared a CFEM domain length of roughly 60 aa (range, 47–67 aa). Typically, in most CFEM structural domains contain eight evenly distributed cysteines that can combine to form four disulfide bonds, which are essential for stabilizing the structure of the entire domain. However, some exceptions were observed, for instance NsCFEM9 and 18, which possessed six conserved cysteines, and NsCFEM4, 12, and 19, which harbored seven. Other relatively conserved amino acids. identified in these CFEM domains include proline (at position 6), asparagine (at position 26), valine (at positions 40 and 44), and leucine (at position 54). The diverse aa compositions of these CFEM domains likely aid in the creation of various structures, enabling them to perform multifaceted functions in plant-pathogen interactions.

Based on feedback from WebLogo, the CFEM domain amino acid sequences of *N. sichuanensis* of NsCFEM proteins are highly conserved (**Figure 1C**). Fungal pathogen effectors are usually a class of secreted proteins that can be recognized by the typical features of containing a signal peptide and lacking a transmembrane structural domain. Our identified five CFEM proteins (NsCFEM1, 2, 4, 6, and 11) as candidate effectors based on these distinguishing features and were selected for further investigation.

### 3.3 Helical basket shape detected in the structural model of the CFEM domains

**Figure 2:**
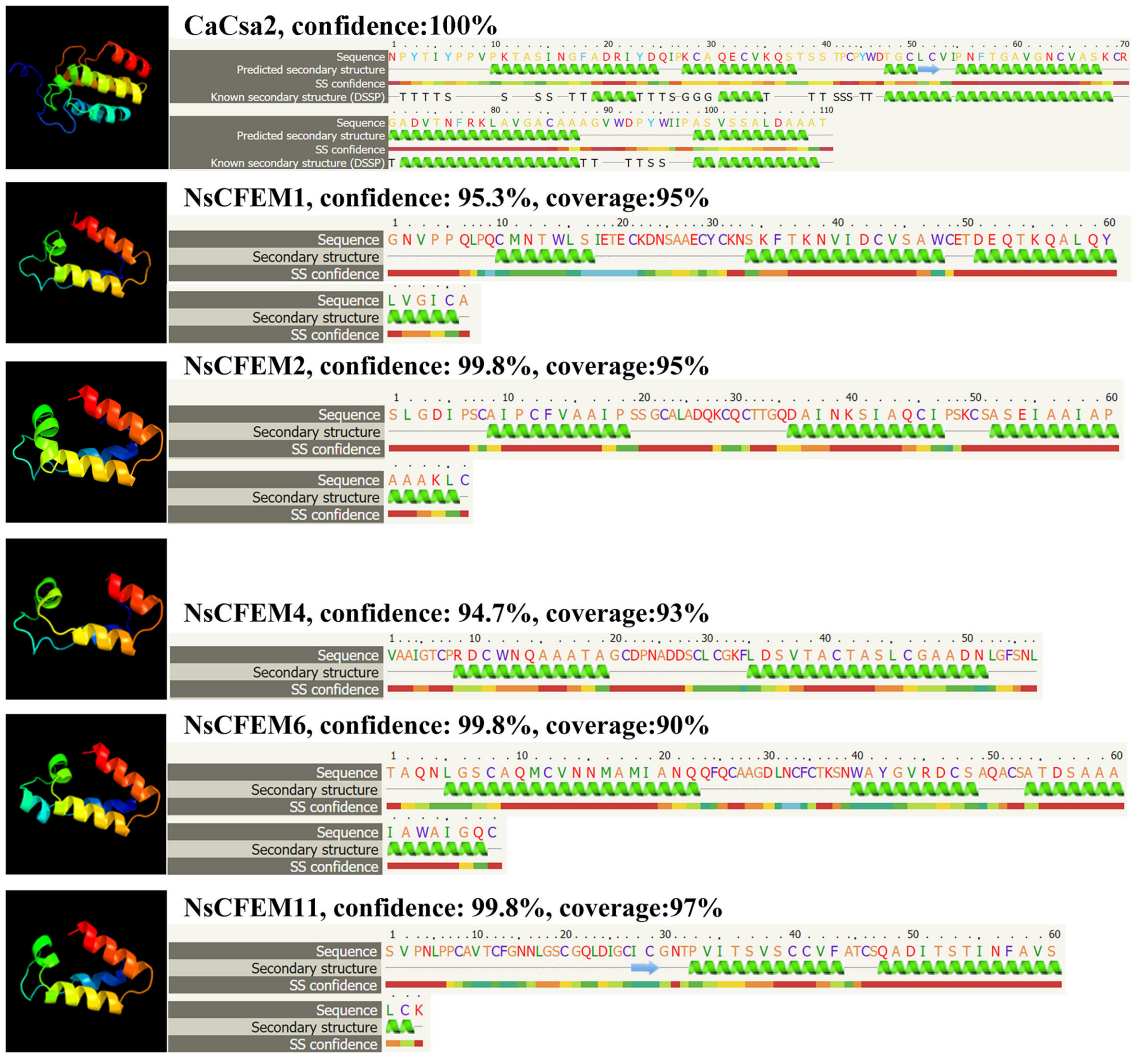
Structural modeling of candidate NsCFEM effectors using Phyre2. Utilizing the Phyre2 Server, the amino acid sequences of the five NsCFEM effectors were modeled based on the 3D model of the CFEM domain from *Candida albicans* Csa2, achieving a confidence level of over 90%. Amino acid sequences of the five NsCFEM effectors are provided and color-coded according to an attribute-based scheme: yellow represents small/polar residues (A, S, T, G, P), green represents hydrophobic residues (M, I, L, V), red represents charged residues (K, R, E, N, D, H, Q), and purple represents aromatic residues and cysteine (W, Y, F, C). Within the models, green helices stand in for α-helices and blue arrows signify β-strands.

We uploaded the sequences of the CFEM structural domains of five proteins (NsCFEM1, 2, 4, 6 and11) into Phyre2 software for homology analysis and protein structure modeling (**Figure 2**). The *C. albicans* protein Csa2 protein is currently the only identified CFEM protein with a three-dimensional stereocrystal structure (Nasser et al., 2016). Compared with the Csa2 protein model, the structural domains of all five NsCFEM proteins were modeled with more than 90% confidence, indicating that these five NsCFEM proteins share high sequence and structural similarity with the C. albicans protein Csa2. The CFEM structural domain in the Csa2 protein contain of six α-helices and one β-strand, forming a helical basket shape with an elongated N-terminal loop serving as a handle. Notably, the CFEM structural domains of the five NsCFEM proteins in this study were modeled similarly to the structure of Csa2, which also exhibits a helical basket shape. Three of the NsCFEM proteins (NsCFEM1, 2 and 6) contained three α-helices. Two NsCFEM proteins (NsCFEM4 and 11) contain two α-helices.

### 3.4 Model quality assessment and structural overlay analysis

**Figure 3:**
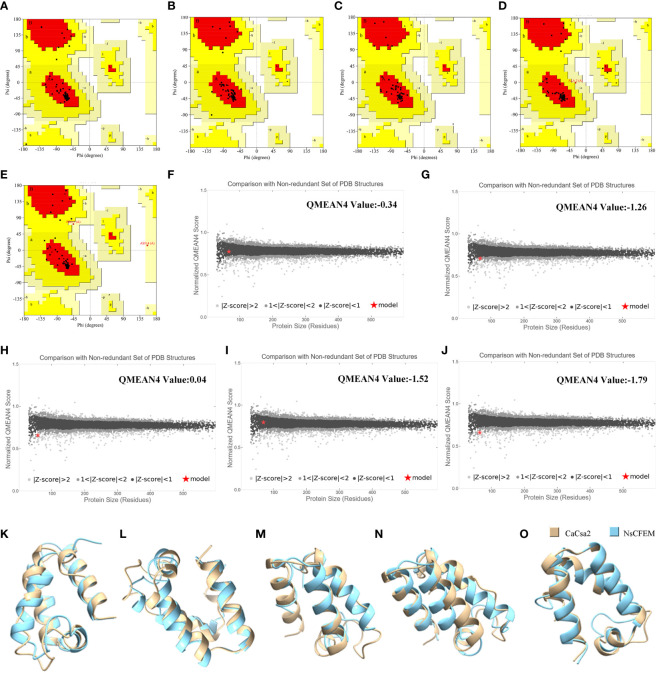
Model quality assessment and structural overlay analysis. **(A–E)** NsCFEM1, NsCFEM2, NsCFEM4, NsCFEM6 and NsCFEM11 models were evaluated by PROCHECK respectively; **(E, F)** NsCFEM1, NsCFEM2, NsCFEM4, NsCFEM6 and NsCFEM11 models were evaluated by QMEAN respectively; **(K–O)** Comparison of NsCFEM1, NsCFEM2, NsCFEM4, NsCFEM6, and NsCFEM11 proteins with CaCsa2 proteins for tertiary structural stacking, respectively.

The tertiary structures of the five candidate NsCFEM effector proteins were evaluated using two quality assessment software programs, PROCHECK and Qmean (**Figure 3**). The results of PROCHECK are presented to the user in the form of a Ramachandran plot, which shows that 93.3% of all amino acid residues of NsCFEM1 are located in the most plausible region of the Ramachandran plot, 6.7% of amino acid residues are located in the second most plausible region, and 0.0% are located in the non-plausible region (**Figure 3A**). NsCFEM2 displayed 92.9% in the most plausible region, 7.1% in the second (**Figure 3B**). NsCFEM4 displayed 100% in the most plausible region (**Figure 3C**). NsCFEM6 had 93.5% in the most plausible region, 4.8% in the second, and 1.6% in the non-plausible region (**Figure 3D**). NsCFEM11 displayed 90.4% in the most plausible region, 7.7% in the second, and 1.9% in the non-plausible region (**Figure 3E**). Overall, a protein model with more than 90% of the amino acid residues are located in the most and second most plausible regions indicates that the structure being evaluated is of high quality. Qmean compares evaluated objects with PDB’s similarly-sized non-redundant structures, returning graphical and quantified Z-Score results. NsCFEM1, NsCFEM2, NsCFEM4, NsCFEM6, and NsCFEM11 models had Z-Scores of -0.34, -1.26, 0.04, -1.52, and -1.79, respectively, falling within the |Z-Score| < 2 range (**Figures 3F–J**). The evaluation results of PROCHECK and Qmean consistently showed that the tertiary structures of all five passed the evaluation criteria of the two software programs, and thus the five protein models predicted by Alphafold2 were of high quality and could be used for subsequent experimental analysis.

UCSF Chimera software was used to compare CaCsa2 protein with five NsCFEM based on tertiary structure using CaCsa2 protein as a reference model (**Figures 3K–O**). The root mean square deviation (RMSD) of all atoms was 0.821 Å, 1.278 Å, 1.181 Å, 1.272 Å and 0.869 Å, respectively, showing a high level of structural overlap, indicating that the tertiary structures of the five NSCFEM proteins are structurally similar to those of the CaCsa2 proteins, which belong to the same class.

### 3.5 Cloning two candidate NsCFEM effectors from *N. sichuanensis*

A total of two candidate NsCFEM effectors (NsCFEM1 and 2) were successfully cloned from the *N. sichuanensis* cDNA. We failed to amplify NsCFEM4, 6 and 11, despite numerous trials. Therefore, we will continue to investigate the functions of two candidate effector proteins, NsCFEM1 and NsCFEM2.

### 3.6 NsCFEM1 effector suppress programmed cell death in *N. benthamiana*

**Figure 4:**
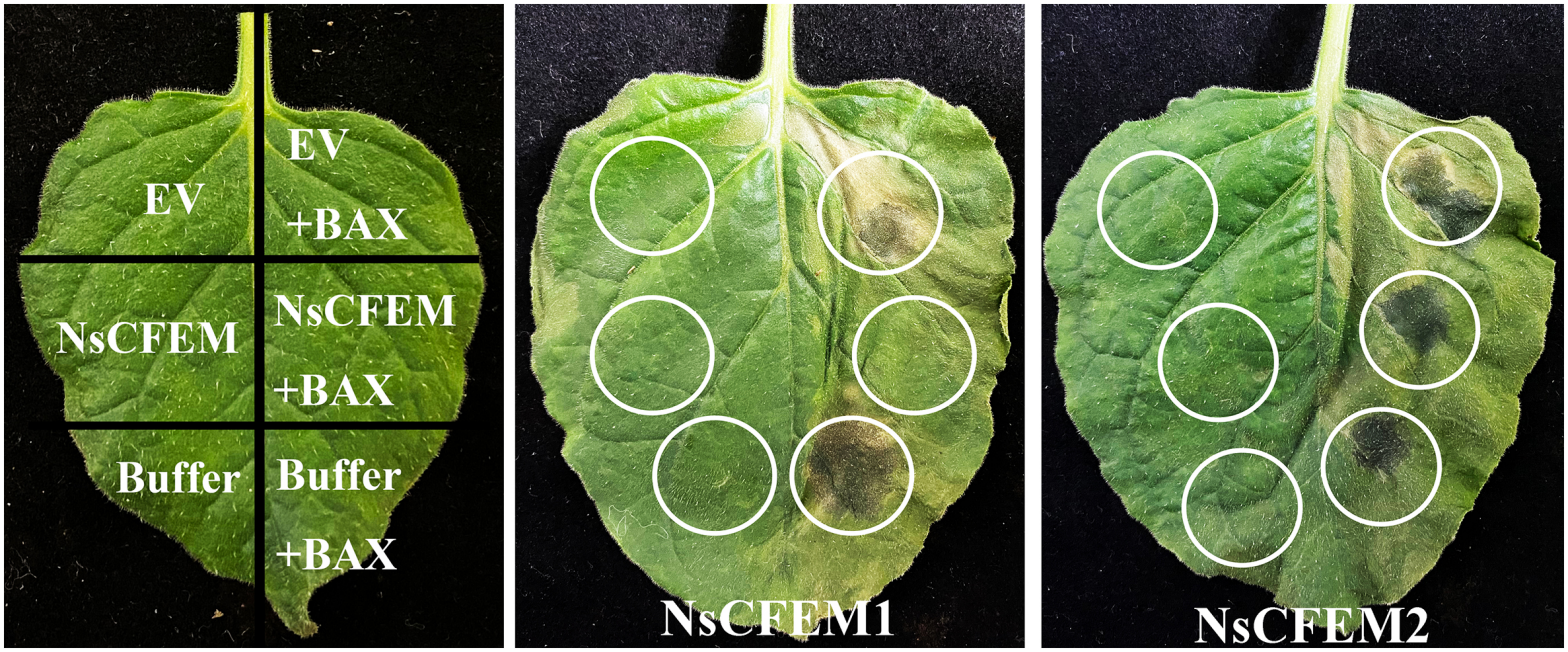
Transient expression of NsCFEM1 and NsCFEM2 effector proteins in *Nicotiana benthamiana* using *Agrobacterium*-mediated method. *N. benthamiana* leaves were infiltrated with GV3101 strains carrying PGR107-Bax, PGR107-NsCFEM, or combinations of Bax and NsCFEM (common infiltration). Bax was the positive control in this experiment. The onset was observed and recorded after five days.

To verify whether the NsCFEM1 and NsCFEM2 effectors induced and inhibited cell death in the non-host plant *N. benthamiana*, we performed a transient expression assay using the *Agrobacterium*-mediated method. Given the similarity in the physiological properties between cell necrosis induced by the mouse pro-apoptotic protein Bax in plants and pathogenic bacteria-induced anaphylactic responses in plants (Jamir et al., 2004), screening for pathogen effector proteins that inhibit Bax-induced cell necrosis induced by Bax was appropriate. The *A. tumefaciens* cells carrying PGR: Bax infiltrated *N. benthamiana* leaves served as a positive control (**Figure 4**). When *A. tumefaciens* cells carrying PGR107-NsCFEM1 were infiltrated into *N. benthamiana* leaves alone, no significant cell death symptoms were observed. However, when *A. tumefaciens* cells carrying both PGR107-NsCFEM1 and Bax were co-infiltrated into *N. benthamiana* leaves, the cell death symptoms were not prominent, markedly differing from those exhibited by the leaves injected with Bax alone. In contrast, no symptoms of cell death were noticed when *A. tumefaciens* cells harboring PGR107-NsCFEM2 were infiltrated into *N. benthamiana* leaves individually. Nevertheless, when *A. tumefaciens* cells carrying both PGR107-NsCFEM2 and Bax were co-infiltrated, local yellowing and necrosis of the leaves were evident, resembling the symptoms infiltrated by Bax alone. These findings suggest that NsCFEM1 not induce cell death in *N. benthamiana* leaves, but possesses the ability to suppress Bax-induced PCD in *N. benthamiana*. Conversely, NsCFEM2 not suppress Bax-induced PCD in *N. benthamiana*. This implied that NsCFEM1 might be a potential effector protein of the *N. sichuanensis.*

### 3.7 Subcellular localization analysis of two NsCFEM effectors

Utilizing confocal laser scanning microscopy, all fluorescence signals were successfully captured in the *N. benthamiana* leaf cells expressing the NsCFEM1 and NsCFEM2 effectors. The green fluorescence emitted by the empty PCAMBIAsuper1300-GFP vector was clearly visible in the *N. benthamiana* leaf cells. Red fluorescence was observed for the membrane and nuclear markers. The results demonstrated that the fluorescence signals corresponding to the membrane and nuclear markers matched the localization of the recombinant NsCFEM1 proteins.

**Figure 5:**
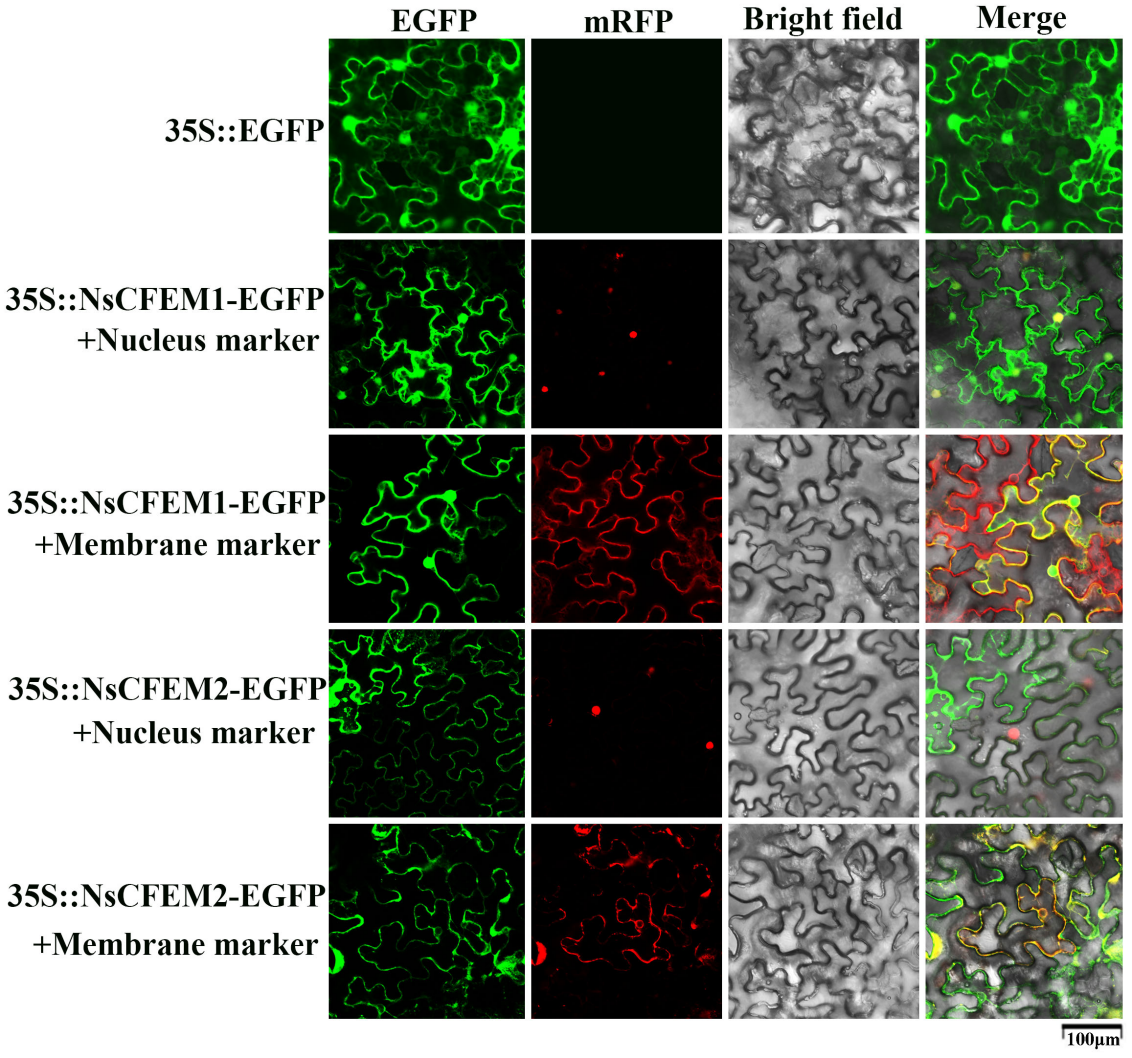
NsCFEM1 and NsCFEM2 subcellular localization in *N. benthamiana* leaves. The 35S: EGFP represents the control group.

Strain GV3101 with EGFP-NsCFEM1 and EGFP-NsCFEM2 recombinant vectors was transiently expressed in N. benthamiana leaf cells, and all fluorescent signals were successfully captured by laser scanning confocal microscopy. Among them, the green protein fluorescence from the empty PCAMBIAsuper1300-GFP vector could be observed in *N. benthamiana* leaf cells. While the membrane and nuclear markers emitted red protein fluorescence. From the merge localization diagram, it can be seen that the fluorescence signal emitted by EGFP-NsCFEM1 recombinant protein corresponds to the membrane and nuclear tags. However, the EGFP-NsCFEM2 recombinant protein corresponded to the membrane marker matched, but not with the nuclear marker (**Figure 5**). These findings indicate that NsCFEM1 was localized to both the nucleus and cell membrane, while NsCFEM2 was solely located on the cell membrane.

### 3.8 Signal peptides of NsCFEM1 and NsCFEM2 effectors have secretory functions

**Figure 6:**
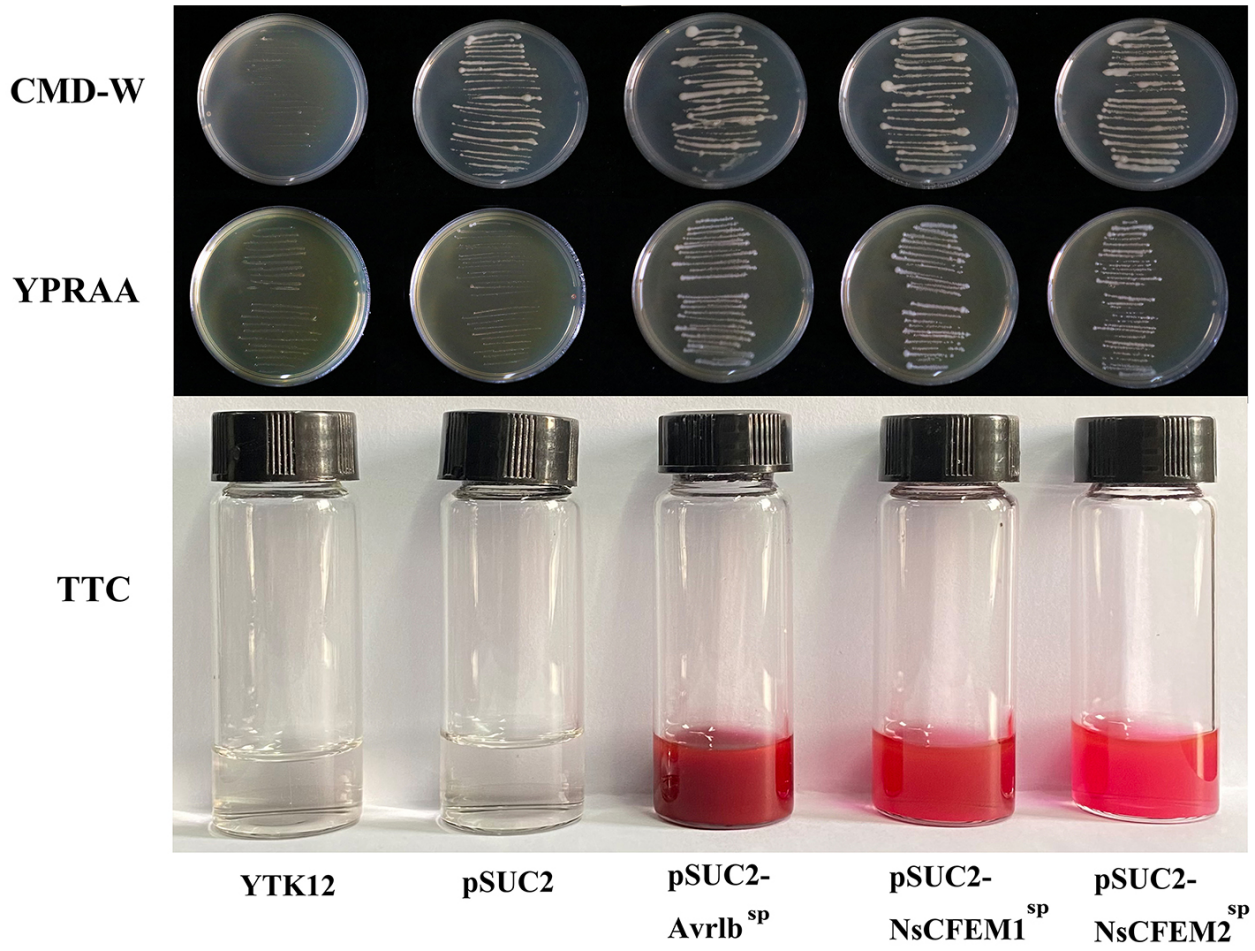
Functional analysis of NsCFEM1 and NsCFEM2 signal peptides. YTK12 strains containing pSUC2-NsCFEM1^SP^ and pSUC2-NsCFEM2^SP^ as well as the positive control pSUC2-Avr1b^SP^ were able to grow on CMD-W medium and YPRAA medium. The results of the TTC assay further showed that YTK12 strain containing pSUC2-NsCFEM1^SP^ and pSUC2-NsCFEM2^SP^ was able to successfully change the reaction mixture from colorless to dark and bright red, which was in agreement with the results of the positive control Avr1b. The negative controls (YTK12 strain without vector and YTK12 strain with pSUC2 empty vector) showed no color change.

The signal peptides of the NsCFEM1^SP^ and NsCFEM2^SP^, were successfully cloned into the pSUC2 vector and transformed into the invertase secretion-deficient yeast strain YTK12. Transformants expressing NsCFEM1^SP^ and NsCFEM2^SP^ displayed distinct growth streaks on both the CMD-W and YPRAA medium, indicating their ability to support yeast growth through invertase secretion (**Figure 6**). However, pSUC2-YTK12, which lacks tryptophan (Trp) in CMD-W medium (containing sucrose), was able to grow even in the absence of secreted invertase. The positive controls (pSUC2-Avr1b^SP^), pSUC2-NsCFEM1^SP^ and pSUC2-NsCFEM2^SP^) grew well. The NsCFEM1^SP^ and NsCFEM2^SP^ transformants efficiently converted 2,3,5-triphenyl tetrazolium chloride (TTC) to the insoluble, red-colored 1,3,5-triphenyl formazan (TPF) during the ensuing enzyme activity analysis. The NsCFEM1^SP^ and NsCFEM2^SP^ effectors’ signal peptides are confirmed to have secretory activities based on these studies.

### 3.9 Preparation of protoplasts and genetic transformation

**Figure 7:**
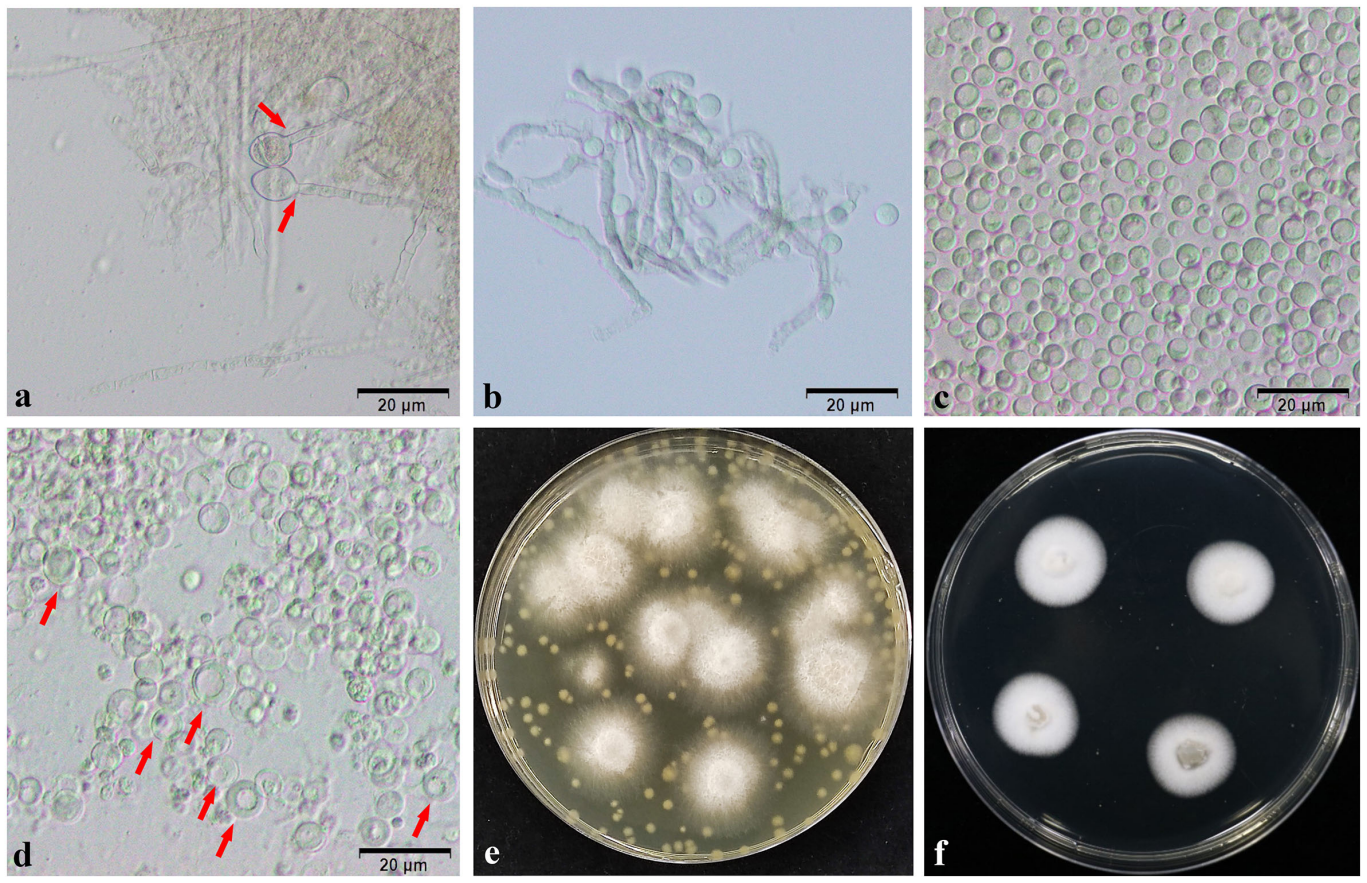
Protoplasts and transformers. **(A)** Expansion of the mycelium tip. **(B)** Gradual enzymatic digestion of mycelium. **(C)** Appearance of protoplasts after washing by centrifugation. **(D)** Revival of protoplasts, with “double-ringed” cell wall. **(E)** Growth of transformed strains for 10 days on TB3 bilayer medium containing the resistance marker. **(F)** Appearance of transformant strains after 5 days of growth. Scale bars **(A–D)** = 20 µm.

In the process of mycelial enzymolysis, the phenomenon of swelling and gradual disintegration, dispersion and deformation of the tip of the mycelium can be observed under the microscope, and ultimately releasing full, round and transparent spherical protoplasts. Following genetic transformation and culture, the protoplasts gradually recovered. After 10 days of culture, many white single colonies grew because of the lower concentration of Hgy in the bottom layer of the plate. In the top layer of the plate, where the concentration of Hgy was higher, a small number of white single colonies can be observed growing on the surface of the plate (**Figure 7**). PCR was used to detect the knockout strains ΔNsCFEM1 and ΔNsCFEM2 (**Supplementary Figure 2**), and complementation strains ΔNsCFEM1+, ΔNsCFEM2+ (**Supplementary Figure 3**).

### 3.10 Phenotypic, sporulation, and spore germination analysis of transformants

**Figure 8:**
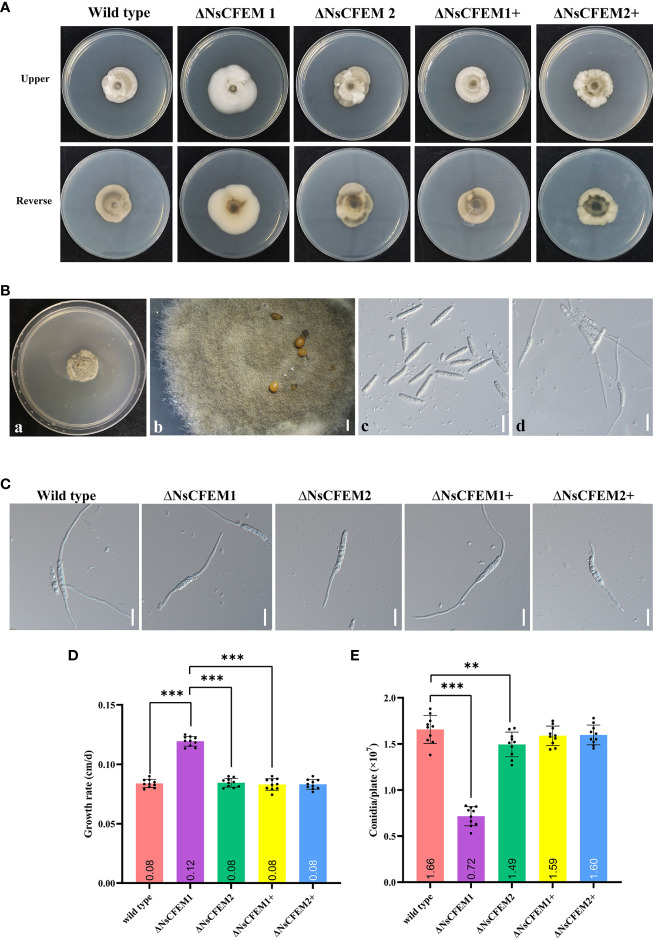
Effects of NsCFEM gene knockout on colony phenotype, growth rate, conidial germination and sporulation. **(A)** Colonies of wild type, ΔNsCFEM knockout,and ΔNsCFEM+ complementary strains of *N. sichuanensis* cultured for 30 d. **(B)** Sporulation and spore germination of each strain (a to b represent sporulation in indoor culture; c to d, spore germination, observed by bright-field microscopy at 40× magnification). Scale bars are 1 mm in b and 20 µm in c and d. **(C)** Spore germination of each strain. Scale bars denote 20 µm. **(D)** Mycelial growth rate of each strain. **(E)** Spore production statistics of each strain. Sample size per independent experiment (n=10). Asterisks indicate statistically significant differences in spore production between the NsCFEM1 knockout strain and the other strains on lactose agar medium containing bamboo decoction, analyzed by one-way ANOVA, *P*≤ 0.01.

The knockout strain ΔNsCFEM1 differed significantly from the wild-type strain and the complementary strain ΔNsCFEM1+ in terms of colony morphology features (**Figure 8A**). The mycelium of the knockout strain ΔNsCFEM1 exhibited a denser compared to the wild-type. Additionally, mycelium produced less pigmentation and colonies grew faster when viewed from the back of the plate. In contrast, the knockout strain ΔNsCFEM2 displayed no significant phenotypic variations when compared to the wild-type and the complementary strain ΔNsCFEM2+. Furthermore, with the exception of the accelerated growth of ΔNsCFEM1, no substantial differences in growth rates were observed among the other colonies (**Figure 8D**). No significant differences exist in spore morphology, septum number, or spore germination rates between strains. The conidial morphology and germination were assayed (**Figure 8B**). No differences in sporulation and spore germination were evident between the wild type, ΔNsCFEM1, ΔNsCFEM2, ΔNsCFEM1+ and ΔNsCFEM2+ strains (**Figure 8C**). However, the conidial production of ΔNsCFEM1 was reduced compared to wild type and ΔNsCFEM1+ strains. In contrast, the difference in conidial production of ΔNsCFEM2 compared to wild type and ΔNsCFEM2+ strains was not significant (**Figure 8E**).

### 3.11 Stress response of transformants

**Figure 9:**
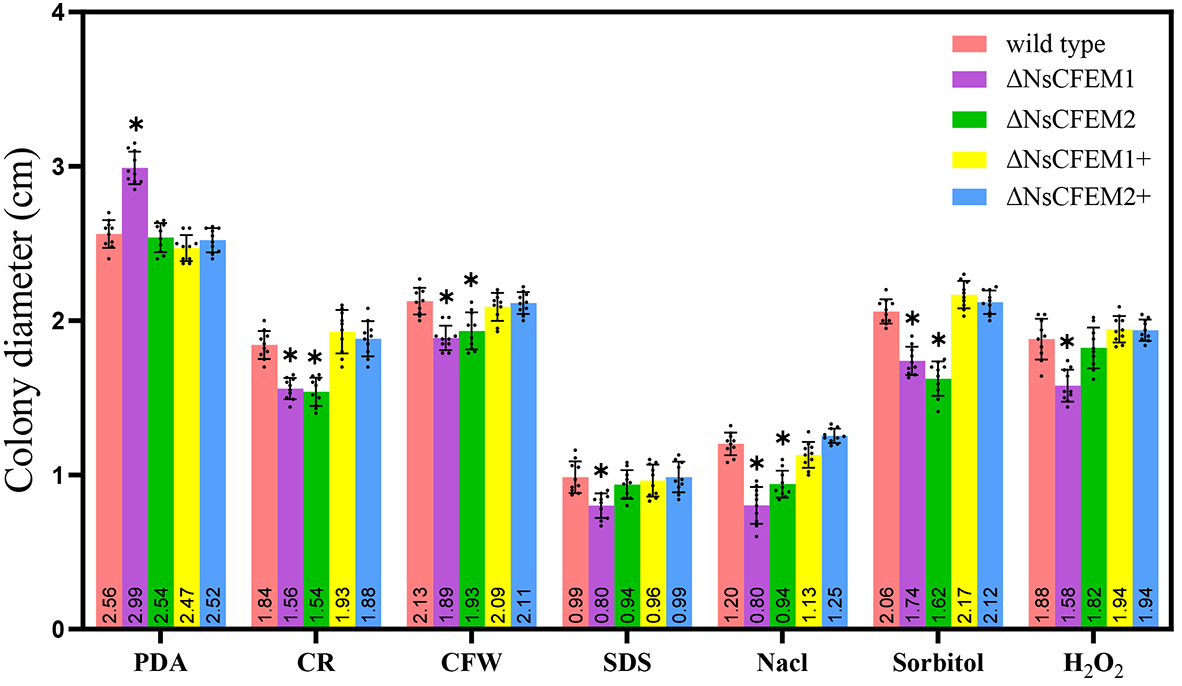
Bar graphs illustrate the mycelial growth of wild-type, knockout and complementary strains under various stress conditions. Sample size per independent experiment (n=10). An asterisk indicates that the difference in mycelial growth between the knockout strain and the other strains under the same stress conditions was statistically significant, and was analyzed by one-way ANOVA with *P*≤ 0.01.

In a thorough investigation of the role of NsCFEM1 and NsCFEM2 in external stress tolerance, we added various compounds into the PDA medium. We gauged the resilience of wild-type, knockout, and complemented strains against salt, osmotic pressure, and cell wall stresses by measuring their colony diameters. The results showed that the tolerance of ΔNsCFEM1 knockout strains to each compound was significantly different compared to the other strains (**Figure 9**). However, we observed no significant alterations in stress tolerance among the wild-type, ΔNsCFEM1+ complementary strain, and ΔNsCFEM2+ complementary strains. These results indicate that NsCFEM1 positively regulates the susceptibility of *N. sichuanensis* to cell wall stressors, oxidative stress, and osmotic stabilizers. Furthermore, the NsCFEM2 may play a role in this regard. The collective findings implicate NsCFEM1 and NsCFEM2 as crucial factors for the growth and stress responses of pathogenic fungi.

### 3.12 Pathogenicity test of transformants

**Figure 10:**
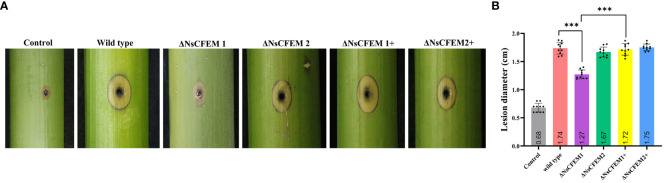
Infection status of bamboo culm after 45 days of inoculation with wild type, ΔNsCFEM1, ΔNsCFEM2, ΔNsCFEM1+complementary strains, ΔNsCFEM1+complementary strains, and aseptic agar blocks. **(A)** Symptoms of infected bamboo culm for each strain. By creating scald wounds on the surface of bamboo culm, mycelial spheres cultured by each strain were inoculated into the wound site. The samples were moisturized and bagged, and the fresh mycelial spheres were replaced every 2 days. The infected status of bamboo culm was observed after 45 days. **(B)** Statistics of the diameter of the injury after 45 days of inoculation with each strain. Sample size per independent experiment n=10. Asterisks indicate statistically significant differences between the knockout strain and the other strains under the same stress conditions in a one-way ANOVA at *P*≤ 0.01.

To clarify whether NsCFEM1 and NsCFEM2 are involved in pathogenicity process, we conducted pathogenicity test by inoculating wild-type, knockout and complementary strains onto one-year-old healthy fishscale bamboo culm. Controls were inoculated with pure PDA medium (**Figure 10A**). After 45 days of cultivation in the greenhouse, ΔNsCFEM1 knockout-infected bamboo culm had milder symptoms, with yellow spots; blackening of a few of the fiber structures of the bamboo culm was observed. In contrast, bamboo culm infected by the wild type strain of *N. sichuanensis*, the ΔNsCFEM1 knockout, and the complementary strains of ΔNsCFEM1+ and ΔNsCFEM2+ showed obvious symptoms, with a larger infected area and an obvious blackening of the fiber structure. There were no significant difference in pathogenicity between the wild-type and two complementary strains, and infection was minimal in the control group inoculated with sterile agar and did not change over time. Statistics on the diameter of infection showed that the range of bamboo culm infected by the ΔNsCFEM1 knockout strains was significantly different from that of the wild type strain and the ΔNsCFEM1+ complementary strain (**Figure 10B**). The infection status of the ΔNsCFEM2 knockout strains was not significantly different from that of the wild type and ΔNsCFEM2+ complementary strains.

## 4 Discussion

During plant infestation by pathogenic fungi, effectors occupy a crucial position in determining the virulence level and host range by either enhancing the pathogenicity of the pathogenic fungi or stimulating strong host resistance by recognizing host resistance genes (Fu et al., 2022). Most recently, an ever-growing number of studies have targeted fungal effectors (Brown et al., 2012; Wang et al., 2022), among which the CFEM family is a class of extracellular membrane proteins unique to fungi. Unraveling their effector functions is of great significance for understanding pathogenic fungus-host interactions. Since the initial identification of CFEM-like proteins in *Coccidioides immitis*, the distribution of CFEM proteins in the fungal community has been further expanded, including *C. albicans*, *C. parapsilosis*, *Magnaporthe oryzae*, *Arthrobotrys oligospora*, *Co. graminicola*, and *Aspergillus fumigatus* (Kulkarni et al., 2003; Weissman and Kornitzer, 2004; Ramanujam et al., 2013; Vaknin et al., 2014). For instance, previous studies have reported 19 CFEM proteins in *M. oryzae* (Kou et al., 2017), 6 CFEM in *C. albicans*, 13 CFEM in *A. oligospora* (Zhang et al., 2015), and 24 CFEM in *C. graminicola* (Gong et al., 2020). Comparison of the lengths of the CFEM structural domains in different species revealed significant differences in the number of CFEMs in fungi (Pendleton et al., 2014; Vela-Corcía et al., 2016), and more CFEM structural proteins in pathogenic fungi than in non-pathogenic fungi (Appella et al., 1988; Fu et al., 2022). The structural complexity, environmental changes, and functional differences in species may be responsible for the increased number of CFEM proteins (Fu et al., 2022). In this research, we have identified 19 CFEM proteins (NsCFEM1–19) in the genome of *N. sichuanensis*. Therefore, we suggest that the CFEM structural domains of *N. sichuanensis* are closely associated with its pathogenicity. In addition, all CFEM structural domains, except for NsCFEM1, 10, and 13, were close to the N-terminus of the proteins, similar to those of *C. graminicola* (Jing et al., 2016). Thus, these particular structures are likely to take multiple roles in the complicated pathogenic processes. CFEM domains are relatively conserved and usually contain a cysteine residue with eight characteristic spacers, forming four structurally stable disulfide bonds (Cai et al., 2022). In this research, the majority of NsCFEM proteins had eight conserved cysteines, excluding the deletion in NsCFEM9, 12, 18, and 19 (**Figure 1B**). The intramolecular disulfide bond structure is regarded as a key factor in stabilizing effector protein structures during pathogen infestation of host plants (Kamoun, 2006). In addition, the aspartic acid residues within the CFEM domains of the 14 NsCFEMs were relatively conserved (**Figure 1B**). Numerous iron uptake systems have been found in fungi, and the CFEM structural domains of *C. albicans*, including aspartic acid, tyrosine, and histidine residues, have the ability to acquire iron ions (Kuznets et al., 2014). Modulation of iron nutrients is critical topathogenesis and can enhance infection rates under specific sets of conditions (Albarouki and Deising, 2013; Nairz and Weiss, 2020). For instance, iron depredation during infection is critical to *Verticillium dahliae* colonizing a host (Wang et al., 2022). NsCFEM proteins might also have iron acquisition functions and may be involved in the life history of *N. sichuanensis*.

Based on the fulfilment of the conditions of having a signaling peptide and no transmembrane structural domains, five NsCFEMs (NsCFEM1, 2, 4, 6, and 11) were screened as potential effectors and two NsCFEMs (NsCFEM1 and NsCFEM2) were successfully cloned. The first step in identifying the effectors was to verify their ability to be secreted extracellularly. Due to the critical role of secreted proteins in the pathogenic mechanisms of plant pathogens, almost all the existing research on plant pathogens has focused on secretomes. Secretory proteins are generally considered essential for successful pathogenesis and pathogen-host compatibility (Liu et al., 2013; Kong et al., 2016; Xu et al., 2019). This research confirmed that both NsCFEM1 and NsCFEM2 were secreted proteins. To interfere with plant immune pathways, fungal effectors are usually localized in specific subcellular locations based on function (Qian et al., 2022). As shown by Pth11 and WISH in *M. oryzae*, CFEM proteins are primarily localized in the outer layer of the cell membrane (Kou et al., 2017; Nazmiara and Subhankar, 2017). In this research, NsCFEM1 was localized to the cell membrane and nucleus, whereas NsCFEM2 was localized to the cell membrane. This suggests that CFEM proteins may act on different parts of the plant body. Effector proteins act as host immunosuppressive proteins and microorganisms control the plant immune system by releasing effectors that help pathogens evade immunity and successfully colonize to establish infections (Chen et al., 2013; Guo et al., 2019; Zhao et al., 2020). However, not all CFEM proteins are involved in virulence. In *Aspergillus fumigatus*, three gpi-anchored CFEM proteins influence cell wall stability yet do not promote infection (Vaknin et al., 2014). In *C. graminicola*, just five of the 10 effectors were shown to have host immunosuppressive effects (Gong et al., 2020). Among the six effectors of *S. turcica*, only StCFEM12 inhibits host programmed cell death (Wang et al., 2021). In the present study, NsCFEM1 inhibited Bax-triggered programmed cell death, whereas NsCFEM2 did not induce or inhibit programmed cell death.

Previous studies confirmed that CFEM effector proteins regulate fungal growth and development (Ling et al., 2015; Arya et al., 2020). For example, when ChEP113, an effector containing the CFEM structural domain in *Colletotrichum higginsianum* was knocked down, ΔChEP113 mutant strains showed an increase in aerial mycelium, a slowdown in mycelial growth and a significant change in morphology (Pi, 2019). Although conidial germination was not affected by BcCFE1 in *B. cinerea* knockdown cells, spore production was significantly reduced (Zhu et al., 2017). In our research, the ΔNsCFEM1 knockout strain had a significantly faster growth rate than the other strains. ΔNsCFEM1 knockout strain colony morphology was significantly altered, the mycelium was whitish in color, and the melanin content was obviously lower than that of the wild type and complemented strains. Fungal melanin is multifunctional and plays biological roles in morphogenesis, pathogenicity, energy transduction, and carbon storage (Zhang et al., 2019). For example, rice blast fungal attachment cells are unable to generate sufficient expansion pressure to penetrate plant hosts owing to melanin defects (Chumley and Valent, 1990). There were no morphologically significant differences between the ΔNsCFEM2 knockout strain and the other strains. Observations of differences in spore production and spore germination revealed that both ΔNsCFEM1 and ΔNsCFEM2 knockout strain were spore-producing and germinable, with no significant differences from the wild-type and complemented strains. However, the spore production of the ΔNsCFEM1 knockout strain was significantly lower than that of the other strains, indicating that NsCFEM1 takes an essential role in colony morphology and spore production.

To further investigate the effect of CFEM proteins upon fungal virulence, there was an observation that the deletion of NsCFEM1 in *N. sichuanensis* resulted in significant enhancement of the cell wall, stress tolerance, and reactive oxygen species (**Figure 6**). NsCFEM1 may play a positive role in regulating fungal cell wall stressors, plant defense-related reactive oxygen species, and osmotic stabilizers. The results of the pathogenicity assay showed that the ΔNsCFEM1 knockout strain was significantly different from the wild-type strain in terms of pathogenicity, as evidenced by the relatively small diameter of the lesions it caused. However, the ΔNsCFEM2 knockout strain was not significantly different from the wild-type strain in terms of pathogenicity. The wild type strain suppresses immune processes by utilizing and metabolizing reactive oxygen species (Fang et al., 2022). Nevertheless, when the reactive oxygen species metabolism was reduced in the knockout strains, either ΔNsCFEM1 or ΔNsCFEM2, host fishscale bamboo failed to suppress its immune response owing to the production of high levels of reactive oxygen species, and the sensitivity of the ΔNsCFEM1 knockout strain to reactive oxygen species was markedly more susceptible to reactive oxygen species than the other strains. In particular, the NsCFEM1 knockout strain was significantly more sensitive to salt stress than the wild type strain. The fact suggests that the effector NsCFEM1 may take an important role in the sodium chloride metabolism pathway, which explains the lower virulence of ΔNsCFEM1 knockout strain than that of the wild type. Thus, NsCFEM1 is an effector associated with virulence. Whereas, there are part CFEM proteins associating with fungal pathogenicity; CfmA-C of *A. fumigatus* may be associated with cell wall stability, and its deletion does not influence the pathogenicity of the fungus (Yakir et al., 2014). Although deletion of NsCFEM2 did not play a significant role in virulence, it showed sensitivity to cell wall stressors and osmotic stabilizers, suggesting that NsCFEM2 may have a similar function. These results provide basic data for studying the function and mechanism of action of NsCFEM effectors in *N. sichuanensis*, and will inform studies of the mechanism of interactions between *N. sichuanensis* and fish-scale bamboo at the molecular level.

## Data availability statement

The datasets presented in this study can be found in online repositories. The names of the repository/repositories and accession number(s) can be found in the article/**Supplementary Material**.

## Author contributions

FL: Conceptualization, Data curation, Investigation, Methodology, Software, Supervision, Validation, Visualization, Writing – original draft, Writing – review & editing. LJL: Data curation, Methodology, Validation, Writing – review & editing. CL: Data curation, Methodology, Writing – review & editing. YL: Data curation, Methodology, Supervision, Writing – review & editing. SH: Data curation, Investigation, Methodology, Writing – review & editing. HY: Conceptualization, Investigation, Software, Validation, Writing – review & editing. SL: Conceptualization, Investigation, Methodology, Validation, Writing – review & editing. WH: Investigation, Methodology, Writing – review & editing. LL: Conceptualization, Data curation, Investigation, Software, Writing – review & editing. CY: Conceptualization, Data curation, Investigation, Methodology, Software, Supervision, Validation, Visualization, Writing – original draft, Writing – review & editing.
