# Supplementary material for: Systematic identification and functional characterization of the CFEM proteins in fishscale bamboo rhombic-spot pathogen Neostagonosporella sichuanensis
Source: Front Plant Sci. 2024 May 31;15:1396273. doi: 10.3389/fpls.2024.1396273 (PMC11176510; doi:10.3389/fpls.2024.1396273)
Supplement: Supplementary file 1 [file DataSheet_1.pdf]

Supplementary Material

1 Supplementary Data

>*Neostagonosporella sichuanensis* NsCFEM1 (Protein ID: PP279572)
MKATYALLAAVVAQEATATWDFFAQPLNCPIRNNNECSDKQKAGFDWSDLKDGDSNFQYGDFDFSKGWKCG
TAHGKRDALTKRTFGGKTIQNKCSKEQPASFGCNKRKDGFSITTIDVSVEFDVEVDLHYKMNDGSLCKQKSVPC
KQSGNTIQNTQCGGAHSVEVYLGSSYKGEKSDCQIGLHHIGFDCTPGQPYTPPAPPAPPARSPSSPPAQPPVKSTH
AVPSPPPASGSCNAGYGGPGCPPAVTSSPSPPAKGPSSSVPLSGNTSPTRPASPPTYETSKISVPSHETPSAPPAGPT
PPPPAGPSSPPPAGSSPAPPSGPSPPPPAGSSPSPPAGPSAPPAPPAVSPPCNGQYGGSCGASSVATSVRQSTGVAVP
SSLPPSPPSQPSSPASPPPKGGNVPPQLPQCMNTWLSIETECKDNSAAECYCKNSKFTKNVIDCVSAWCETDEQT
KQALQYLVGICAQYVPQNPGLVGDCPKGTFNPPTAAPPAPAPPAPIGGGSSAAAVPSPAPTPANPAPQQPVTTIV
YSSTTVTVPMVHFTTETPVAGATPTAPVGLIPGTAPPSTPAVTTGAPGAPGAPYPTPSTLRPVTVLPGTGTGTGAL
RPSSPAQFTGAASPLNIDAKHAFLGAALAFLAL
>*Neostagonosporella sichuanensis* NsCFEM2 (Protein ID: PP279573)
MATSFRIHHFFVLLLPATTTATLAFKERTRIQITVKMQFSILAIAASSLAVATAQSLGDIPSCAIPCFVAAIPSSGCA
LADQKCQCTTGQDAINKSIAQCIPSKCSASEIAAIAPAAAKLCQAAGVTLTNLPSGTPAASSAASSAAASGSAAA
SSAAASGSAKVSSALASASAAASSATSASRNATLSATQTPPTSTGGAVANAVNIGAVVAGLAVLAL
>*Neostagonosporella sichuanensis* NsCFEM3 (Protein ID: PP279574)
MKRNKIMRNERQVHEHAFSTQLDNRVVYQENLHLEPNFRATMMYDVVVSSVGPALSSRNILHQLNQDLRFHH
QSSSHIKMKFALAGSVIALAAVASAQLDNIPSCALNCFLGPLGSDGCSALTDFKCHCQKGAELVAKVTPCVQGA
CSAADQSKAVAAVQKTCQDAGIPINVPTPGASSAASAASSAAASSAASAASSAASAASSKVASATSAAASAASS
AAGAASSAASKVSSALASATGNGTAPTKPTATQFTGGASQATQAAALIGAAALAMLAL
>*Neostagonosporella sichuanensis* NsCFEM4 (Protein ID: PP279575)
MKVSVSLVAVVFSAVSTAVASPATPTKPLGPGPGPNVAAIGTCPRDCWNQAAATAGCDPNADDSCLCGKFLDS
VTACTASLCGAADNLGFSNLLNPESSSYSQQQQQQPSTSPADSHMATATVSLMAPLLQNAPQQSAQEETRQD
LPRPYKCPLCDKAFHRLEHQTRHIRTHTGEKPHACTFPGCTKRFSRSDELTRHSRIHNNPNSRRGKGQHAAAHQ
AAVAAVQAGLMEPGSSLAHMMAPPSKPISRSALSSNIGSPNVSPPHSLSNYSPNMGNDLAAYRHGGHSNSTSPN
GLSRNMDLLADAASRLEQRPSHHSTRHHITSGYHPYNHRLPGLSQYAYSSQPMSRSHSHEDDDPYAHRMTKKS
RPGSPMSTAPPSPTFSHDSASPTPDHTPLATPAHSPRLRPHGLSDLQLPHLRHLSLGQNFAPALAPMEPSTEREPA
YVPSKNVGGLRISDIMSRAEGAQRKLPVPQVPKVAVQDLLNGPPNGGFSSGNSSSNPSLAGEDLAHRT
>*Neostagonosporella sichuanensis* NsCFEM5 (Protein ID: PP279576)
MRAPSSSSAMSNNFSLFMPVFSMSIQASQSASQHLYIRKTPLTALKLPQCALICYLNALLSDGCANEVDFSCHCSS
GNIISKSKGCIAEGCNDADQADGISKVEAACKAMEGGGGGGGGEVCAGNGNGSGNGIGSGQASRSSSAVASSA
LSGPPRTSAQAKSAPKSNNTSTTYFNTKLFLTERDCPANNPYNDKVFGTLAVKSIESITNTFGTLPSIRLFVVSVTI
VAVTIFFALGWYIRHLKRDLRATQAPTGISDDAWRTHMSAASAAAAPTAINRIRSWSSNGSRRERRSSREFRDGP
VSPLSPRSMSKITTVTDNGYGVLKKKRGNVLSIVVEGSEEDASSLMERGIREPVPGQREGLNAPLELDGERTGVQ
EMLLAITPRNRSWDRDRDSRRPRTGD
>*Neostagonosporella sichuanensis* NsCFEM6 (Protein ID: PP279577)
MKSFAILAVAGAATAQNLGSCAQMCVNNMAMIANQQFQCAAGDLNCFCTKSNWAYGVRDCSAQACSATDSA
AAIAWAIGQCAGQASGAASGAASGTAPAAIPILSSALAGASGTAGSAVASLTGPAGSAITTQPLVATLTNSAGSV
VTTTTGFSTIFSSAASGAASAASSAASAASSAASGASSAAASAASSASGAINSAASSAASAANSAASKASSAAAS
ATSGAPSQGAAAKVTGLPIAAGAGLAALFFLRAHQADISFKLLAITGALSELSLLLCLATDIPLLCSRDVEFISERI
GACKFIFEKVKGAESSLGDYMLSGSHVRGSRVPASLNAFNKGEEVEPLRILQSCLHEILRVTIVARKAFLDTRAA
TFGDEMLEKVRLDGALAHAALNLRDSAFTLHTTDNIKATATQSTLLQTQHHTPQLQTRSILGDNFEHHTTPWDL
WGDMGSDVSTPQQTPVSEAPGSMVDPEQNMPEPKQAPGESADNTSDENPAAKSAKPFQTSADLSLARQSTTIDP
DVASGIDDLTLTFSAFTLSPGHRLQAYILQPKFSFEQSSLFQRYIRWTIEIDAIPMDENAIITHINCITPFYNVFSTLA
ELPPYQFIPIQDRVLRSGGELVSVYPGKVKEEHTEMGIALVNTVMFVIRIPIKQDNDTNPTSQGLFPVAKPGAVFG

PSAPGPLQRQGVAAHSSGAKACEDLTFYKEPYAGGVDEFDSIMSSEQYRNANISHEELRLADYKAGLKGPVAEP
TNVPVASSQAEPASTDPAVSSQASGRSTSLVSSGHKSDPSSSGPAHRELTEGEKLFAKKFKVLSSKPTIASFPTGSS
APPGESNAPRGLWGASSSLPQTSSKPFDFAAAANTASGNPTEAKPSTQLFGKKAKVTQPIHPFAPHASSTPSGFVF
GKAAPSSSLASQSRIPNPFRDRKTTQNNHKPSETGMPESSKSGEQSEMPSAATRPSTPPPWACKHCGESLVFHPA
AEQDAQICDICKGLEDTKCWLCVKRIQAGATAVVKIDDIAKEDTDAGDAVDKGKKGGKGKKNKRK

>*Neostagonosporella sichuanensis* NsCFEM7 (Protein ID: PP279578)

MKVLGFLLSASALAPTILAQEEFLQRLPSCSAKCVVATVPTSKCSPTDFTCLCADTTFMRNAAACNAANCTVVE
VLKATNETYAACGVPPRDHSGTLIGVIASIGSLALLMVVLRLADRAFSHQAQLGWDDLLIGLSGLVSIGMNTPVI
VAGRLGFGRDIWTMKPTNITESLKWLYFAYFMYMMAESLCQLSILAFYLRIMVDRHLRKVVWVCIVMVAGFG
LGNTIAMIFQCTPIRFFWDGWKGEMKGYCGVDVRMFGFIKGAIQIFMDIFILSLPLPMLWKLNMSSKKKLQIMS
MFCVGFIITIVSCLRLWSFVEFAHTQNPTYDNTPGIWWCATEANLFTIVACMPAMHALAHKFLKNFRNSTYASR
GRSGQYGSTPSKGSYVRQGSEKRSNAVVPFGHIKKSTNVDITRTERSDSDVELVTRLPRT

>*Neostagonosporella sichuanensis* NsCFEM8 (Protein ID: PP279579)

MRVRTLITVIVSLFVFANSGVSALNTGLREFTLADMPACGMQCILLTVPVSGCSFEDDVCVCQNKELGASLASCL
LSNCTMADTLDTARVQAKLCNLPNESRRTGVYLVSVIVHALTVLLVILRFVEKTLSKRFSKDDWVVGAALLLSG
VPLALVLAMTSLGFGEHLWNLEDGRLLPILRYFLIASSTYVLVLGLIKVSLILFYIEIFDTPRFRIAAYIVLAYICVS
SVVICLLTLFSCTPVQYFWNRDIKGKCLNVQALAYANSASAIVQNIILLVFPLVSIRNLRMRRCKKVAVAFMFSV
GIFGCIATIVRLQTLLQFKISIDPTWDYVPAVIWMVLELAAGFACVSMPCIRILAVRILPARFREPLSQITQPSKGRS
YPTQDSNMSPRPRQWKTPPSWISLTKPHDSGKGSAARDNFFSRMWSRPSSIASTHRHMRQGSRKLESALSNYAE
STVAVTRPPFCETRNDRNEGVMKHTEQMELLQAPKMNQSKRSSTQSNVSRDSQLTALPQIGKIGILPNQSFSTLN
IGINSRERFRNTGGTNLYKTYQ

>*Neostagonosporella sichuanensis* NsCFEM9 (Protein ID: PP279580)

MKELPKSSCATNLTSECLCSNTELNAAVAVCASKTCTVYELLQTKNVSSKGCGVPVRNKGTKFTIIGITGIVVAA
LAFALRMAASLGKRGRQMSWDDLTMGIVLALAIPPAVFAPTLVQNGLGRDIWTLNAEEITNVLRFYFYGEIFYV
VALGISKISILFFYLRVFPAKSFRMIIYALMGLSLAYTIAFLFATTFQCTPVSQAWTQWDGLHEGTCNNIHLQGWI
AAAINIVLDIVVMILPLKQLAGLQMNMVKKLMVMAMFSVGLFVVVVSVIRLESLIHFANTKNVIWDYFEAGYW
SLIEIDVSIICGCMPAQSKLLARAWSKIKTTIHASKRTSDSSSSGYQSKSGGTHSSAPNQAKAATVKIKPKTGDEG
DFVPLVNMENNHGAASLAGSAEHEKSSEDCIMRTTSVELSSNSEALTAASMINQSLPNWSMGKPAMRRDHV

>*Neostagonosporella sichuanensis* NsCFEM10 (Protein ID: PP279581)

MKRNKIMRNERQVHEHAFSTQLDNRVVYQENLHLEPNFRATMMYDVVVSSVGPALSSRNFLQRIMPQEGLLPT
ASLDDVRQSAVSTNREKLHVTVSQSEPMIQLFAHHHVPVPCRRFAGPSTVSLFVVGMLRLSRIGVELYLFQNLPQ
IDRGKPGQERPQHILHQLNQDLRFHHQSSSHIKMKFALAGSVIALAAVASAQLDNIPSCALNCFLGPLGSDGCSA
LTDFKCHCQKGAELVAKVTPCVQGACSAADQSKAVAAVQKTCQDAGIPINVPTPGASSAASAASSAAASSAAS
AASSAASAASSKVASATSAAASAASSAAGAASSAASKVSSALASATGNGTAPTKPTATQFTGGASQATQAAALI
GAAALAMLAL

>*Neostagonosporella sichuanensis* NsCFEM11 (Protein ID: PP279582)

MRFSIATTVAALSATAIAQSVPNLPPCAVTCFGNNLGSCGQLDIGCICGNTPVITSVSCCVFATCSQADITSTINFA
VSLCKSNGVNVNTSPSCPASGSASASASTQSGSAAPSASGSTLASGVSGVTGTSTPAASGSATTARSSAEAPLPTA
GVGYSLGMVLADVWMRLNKEKGEQHVVVAVLSHHLVQDLELWRGLDAVRVLTRQPANALDHFSTKQPPHN
DNETRRPTSPRRNHG

>*Neostagonosporella sichuanensis* NsCFEM12 (Protein ID: PP279583)

MFNSAIPKRPGESPQIERWRKLPAKPLPWRGYRPRPPLTLACFMKELPKSSCATNLTSECLCSNTELNAAVAVCA
SKTCTVYELLQTKNVSSKGCGVPVRNKGTKFTIIGITGIVVAALAFALRMAASLGKRGRQMSWDDLTMGIVLAL
AIPPAVFAPTLVQNGLGRDIWTLNAEEITNVLRFYFYGEIFYVVALGISKISILFFYLRVFPAKSFRMIIYALMGLSL
AYTIAFLFATTFQCTPVSQAWTQWDGLHEGTCNNIHLQGWIAAAINIVLDIVVMILPLKQLAGLQMNMVKKLM
VMAMFSVGLFVVVVSVIRLESLIHFANTKNVIWDYFEAGYWSLIEIDVSIICGCMPAQSKLLARAWSKIKTTIHAS
KRTSDSSSSGYQSKSGGTHSSAPNQAKAATVKIKPKTGDEGDFVPLVNMENNHGAASLAGSAEHEKSSEDCIMR
TTSVELSSNSEALTAASMINQSLPNWSMGKPAMRRDHRTRDVDDQSTDRPDLASSFQAPSSVHLSARLMGYHV
PLKTALRMQLKLLVLVFVPSSIITNAFPTISTPWDSLIADKLALDIEESLAKDKRSNINPQQPIDITGEHAFRAPGPT
DQRGPCPGLNALANHAYISRSGITSLTEVVTAINQVMGMSIELALILGVMGTVWTGNPLSLNPGFSIGGTSNGNG

DDSSNPTGNLMGILGKPRGLQGAHNWLESDASLTRNDLYVTGNAWTMNMTLFRDFYDRADAGGVISMDLLAS
QAARRFDDSVASNPNFYYGPITGMVSRNAGYMFLGRLLSNHTAEHPEGILTQDVFKKFFAVYTNTQGHLEYRS
GHETIPENWYRAPVDYGLVQLNVDIVGWIKKYPVLGSVGGNTGKVDSFTLLDMRNVSRGALNAASLLENNNL
LCFAFQLMKTFAPNSLSSLLSTIEKPLNLVTSALSTPLLSLACPAFDDMTKSGDSLWETIQAKFPGASQAKSSL
>*Neostagonosporella sichuanensis* NsCFEM13 (Protein ID: PP279584)
MALRRPGYTLTFPAFLHAGYHVSTPCFGGKSSPHDGGVFNQVDCPRRESLGGKQTSINPILNFIHSNLPSTYIMKA
STILSTIALLLASSVSAQTACDPVASAVPTCGVPCIQSAAADFGCTGTDYRCRCNNADAIQSKATNCVITGCGVTT
ALQVQASASAVCACVATAPARL
>*Neostagonosporella sichuanensis* NsCFEM14 (Protein ID: PP279585)
MTGCASPGMNPLSGSTVANHESIMGWGRAADREHAAAISAKATAELGIAFATVLLVLYTTLVSAQEALPLCAD
KCYQLANLQADCTFGNNDCTCSNAAVINELEGCWATSCSMRDFLHAKNTTATVCERPVRDKSYVLVYASTICG
SFALLAVLMRVFVAVRQNSFGYDDLCACLASAMAVPNFIGLAISAKEGLGKDMWTLTPEEIQKCLRQIYICQNF
YFWCSGFTKLCFLFFFLRIFPSQGARTWCFIGIAISLGYAAGFGFPMTFACWPIPAIWTAWLGESAAPDYCINQNIF
YYCAAAVNIASDVLIALIPIPQLWILRFTFKKKMLLLAIFGVGFITIIVSCLRVQSLTQYANTKNPIYDTLSSATWS
VIELNVGVFCACMPAFRRFLAHTMPNCFGSIEDESETLAAATGASSKNVRFAAKKGNKKRSTLPTSLFHTTAKTL
ETQSESTKHDDDELHLVEIGQGDYVSRPVDELYWHAQLFPSEVILQSTHVPPLPRSQADYAEWQKLMREQNAE
L
>*Neostagonosporella sichuanensis* NsCFEM15 (Protein ID: PP279586)
MPVTSRSPWLPSDFSGASNTSRATAPLFSAIPNAILLCDGEFRSLLHLQIRALAELGPTKARESSANELVDANGCV
FCFLLQLHRSFEMKVLGFLLSASALAPTILAQEEFLQRLPSCSAKCVVATVPTSKCSPTDFTCLCADTTFMRNAA
ACNAANCTVVEVLKATNETYAACGVPPRDHSGTLIGVIASIGSLALLMVVLRLADRAFSHQAQLGWDDLLIGLS
GLVSIGMNTPVIVAGRLGFGRDIWTMKPTNITESLKWLYFAYFMYMMAESLCQLSILAFYLRIMVDRHLRKVV
WVCIVMVAGFGLGNTIAMIFQCTPIRFFWDGWKGEMKGYCGVDVRMFGFIKGAIQIFMDIFILSLPLPMLWKLN
MSSKKKLQIMSMFCVGFIITIVSCLRLWSFVEFAHTQNPTYDNTPGIWWCATEANLFTIVACMPAMHALAHKFL
KNFRNSTYASRGRSGQYGSTPSKGSYVRQGSEKRSNAVVPFGHIKKSTNVDITRTERSDSDVELVTRLPRT
>*Neostagonosporella sichuanensis* NsCFEM16 (Protein ID: PP279587)
MKATYALLAAVVAQEATATWDFFAQPLNCPIRNNNECSDKQKAGFDWSDLKDGDSNFQYGDFDFSKGWKCG
TAHGKRDALTKRTFGGKTIQNKCSKEQPASFGCNKRKDGFSITTIDVSVEFDVEVDLHYKMNDGSLCKQKSVPC
KQSGNTIQNTQCGGAHSVEVYLGSSYKGEKSDCQIGLHHIGFDCTPGQPYTPPAPPAPPARSPSSPPAQPPVKSTH
AVPSPPPASGSCNAGYGGPGCPPAVTSSPSPPAKGPSSSVPLSGNTSPTRPASPPTYETSKISVPSHETPSAPPAGPT
PPPPAGPSSPPPAGSSPAPPSGPSPPPPAGSSPSPPAGPSAPPAPPAVSPPCNGQYGGSCGASSVATSVRQSTGVAVP
SSLPPSPPSQPSSPASPPPKGGNVPPQLPQCMNTWLSIETECKDNSAAECYCKNSKFTKNVIDCVSAWCETDEQT
KQALQYLVGICAQYVPQNPGLVGDCPKGTFNPPTAAPPAPAPPAPIGGGSSAAAVPSPAPTPANPAPQQPVTTIV
YSSTTVTVPMVHFTTETPVAGATPTAPVGLIPGTAPPSTPAVTTGAPGAPGAPYPTPSTLRPVTVLPGTGTGTGAL
RPSSPAQFTGAASPLNIDAKHAFLGAALAFLAFARATRRQHGQTMSFVVDQVRRIDSQLDRLQLATTRPGGSF
STLAAEEAHNPAKAARIAHLQNLIKNLSTTASSKSDLVPSYRILETLQRADLSSNCSTCTQWFAQDTDGKSDDAD
EAERGEASYEHELEWLLLSKATTQAYGQVLSTILEQTIPLEDDIWYWDDILSTYRFAGLYSIQTSPIRLWKWGQEI
FHDVRSRGGHLADGWTQFYGLVKDAVQERSIANIQQRVVSPLALVRNEGRRKRAALRKIRLVNANALGILLGE
GLSNESMQDDGIQSPGLFGTQDHRHRWKSTISKSIALMDAVIQSVNTAELSVDKFDDTVASITQEDQYYELNEPA
NDRTATTLKPGEVAERLQYLLKQALPTYLTNFNTVTKENGRPSRIVRYWLPATILLVSSTTIFRIAINRKEEILAW
VRDLGQTVIDFWTNWVVEPTKKVIGTIRHDEDSEVSIMSKRSLQSDRDSLERMVVDFATKNPEGPAPTEAQIADI
QAKVREGDLTPVLKSYEKEIQSPIKGAIMGNLASALLIQVQKTKVDVEVAMSGIDSILKSQELLFGFIGLTPGVLV
SVGVFRWFRGLSSSRKGVQQWARQGKLLLILRNIDRILTSATPTEFGEVSYKDHGLLLCEVHLLRQAASGILPRRI
FHDFLVEVNELVDVRSGLERQQKVVERIRWAYSKWLTATLQRQRPPGTGQRSPDTDIRALSHPQPCYRPRPPRP
PPPPPPQHTPVRRLDSRPPPAYLVPLARTFQFLTAQPSTGSTHTNMAGTRNYDFLVSLARACNFPPLPARNSTANT
PPAQIKLLLIGDSGVGKSCCLLRFSEDSFTPSFITTIGIDFKIRTIELDGKRVKLQIWDTAGQERFRTITTAYYRGAM
GILLVYDVTDERSFNNIRTWFSNVEQHATEGVNKILIGNKCDWEEKRAVSTEQGQALADELGIPFLEVSAKSNIN
VDKAFYSLASDIKKRLIDSARTDQASGPKVDVGSADNANGGGMGGNAIPSSKPLLGHDYAVSEEDAHRRG
>*Neostagonosporella sichuanensis* NsCFEM17 (Protein ID: PP279588)
MAILRTTAAPAAMVLKGSQPVYAVVRDSKSLTYAGKATPSASQPMGRQTQTLGEKVEIQSYAFVLEENRYFVK
LLHRDVPIRPEKAILQRSEAKSPPVEIPTTKSLRKGANSFNMHPIAFLLGGLPLLFIAGTGVAQATLPQCAATCFES

AVGNQTTCTPTDVPCICSSQPLNAAIQGCVMTTCTLKEALVAVNTTNAMCGIPIKDRTNYLIGVNFTFGIIAFLAL
GVRMLVSVQRHIFGADDLCAVVAFAFAAPVTAGQIACGYLGFGKDTWAVSAENIYKIMKIVYLNQLSYFISSTA
SKMCFLFMFLRVFPDARMRRFVYAGMGLSLLFSFAFGLPMVFACTPVSAVWTSWDKETPYKRCINQNVFWFVT
AAYNIAVDVYIVSIPIPELLKLNLSTRKKLMIVAIFSTGIITIIVSIARLWALAQYGSSTNPIYDNMLSGIFSPLELNV
GIVCMCMPAFRRFVGRFLPSCFDSSSKYREHREHEEGSPVPIARVSSGKRSGAKKNSIGASLLETTVKSVDTTKD
NGEDELRLMELRRTNQGEYVSNPDKKKKGIRVRR
>*Neostagonosporella sichuanensis* NsCFEM18 (Protein ID: PP279589)
MRIWQFLLPLLTWITFGLAQNTELISVISSLPQCAQIYLAKSVAASTCNITDVACICANRELQTQVEGCVLQSCTL
RQSLGENSRARISPNIELINVPYSYEELRNEGDKARISNIVVAVVAVACALSRLIYKSAFSVGGLEWDDWSVLAA
VLAGVPSVVMIDRGLVRHGLGQDVWTVPFDHVTNFVRYLYILEILYFLQIALIKLALLFFFLRIFPREQDGKCINI
NALAWSNAIISIVLDVWMLALPLYEIFQLQLSWRKKISVAFMFFVGTFVTVVSALRLKSIVHFATSLNPTWDQTD
AINWSNIEINIGIVCACLPSLRVIFVRMFPSMLATTKGATNNYSRYGNRSQGMGGSSMLKSGHGKSQNGGSAVD
NKITYTKTFPVQYTDSDETSLVQMDDFGGKTPKSNIATFSIIRISRAVLQPMGSLPTTTPDTRSLPRILCLHGGGVN
AAIFKAQSRSLIRDLQHSFRLVWADGPFLCDPHPDIVSVYGSYAPFRRWLRWLPEHSEIDDESCIEEIGYAMRTA
MQDDDREGGTGEWVGLLGFSQGAKLSASLLLEQQAREAKARKEKKEIEIGLTGVPGIHWRWAIMLAGRAPLSN
LNPQILKSTALVSAGSLSEGFEFCSKVDEEAILRTPTVHVHGMADAGLCLHRKLLKDYCEEGTSTLVEWDGAHR
VPLKTVDVDPVLKAIYDVAESTGIRVVRTV
>*Neostagonosporella sichuanensis* NsCFEM19 (Protein ID: PP279590)
MRRGVTSLGSSFTYMLPATDLQTTNHYITLSTRSSPLLLSPNILKQAQQHHLTFKSLPIDFDTYPLPSSPSPALPLL
RTWAHALYVLQLLIGALITIERVIVPVIRANVPQQMCVNNMAMIANQQFQCAAGDLNCFCTKSNWAYGVRDCS
AQACSATDSAAAIAWAIGQCAGQASGAASGAASGTAPAAIPILSSALAGASGTAGSAVASLTGPAGSAITTQPLV
ATLTNSAGSVVTTTTGFSTIFSSAASGAASAASSAASAASSAASGASSAAASAASSASGAINSAASSAASAANSA
ASKASSAAASATSGAPSQGAAAKVTGLPIAAGAGLAALFFLITHARTQACREAIGKRHVIPNTRHVTIHERLDINP
RRVAIMQGRGRHDKGDN

2 Supplementary Figure 1

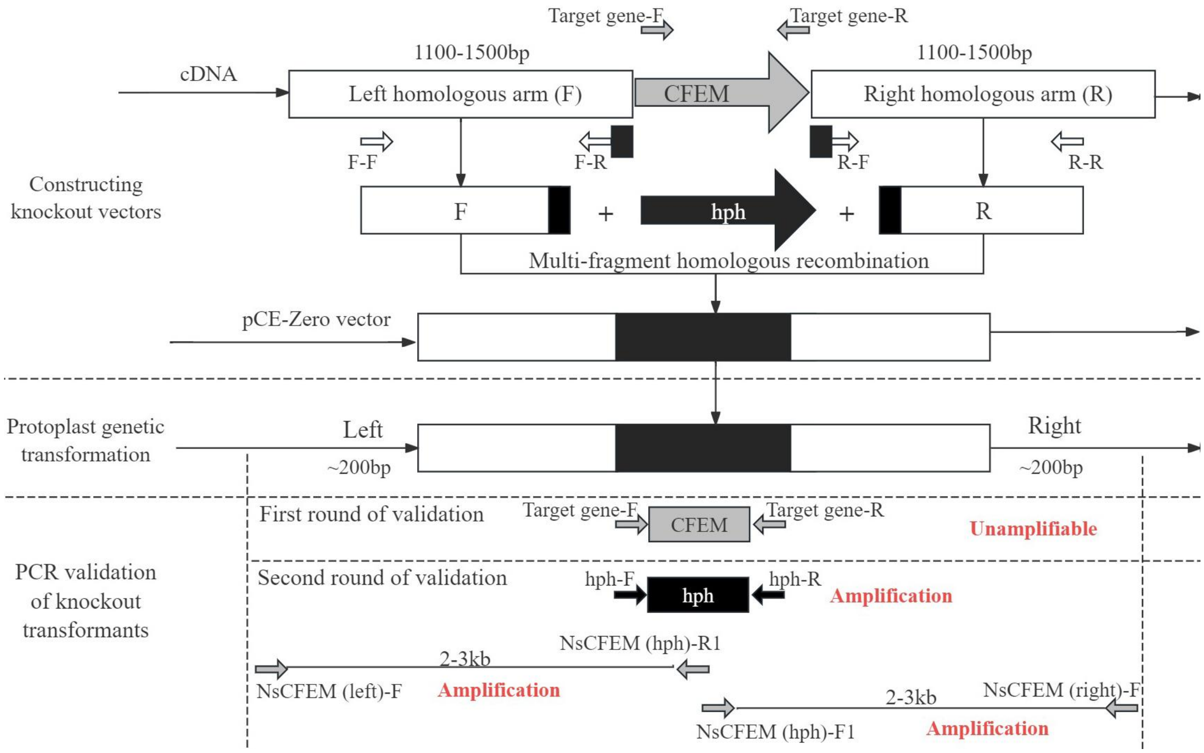

Supplementary Figure 1 Construction of knockout vector and RCR validation of knockout transformants

Note: A: Electrophoretic detection of homologous arms of target genes and *hph* fragments; B: Electrophoretic detection of fusion fragments; C: Electropherogram of the first round of PCR assay for NsCFEM1 and NsCFEM2 knockout transformants (Red numbers indicate positive knockout transformant PCR validation results); D: Electropherogram of the second round of PCR assay for NsCFEM1 and NsCFEM2 knockout transformant.

3 Supplementary Figure 2

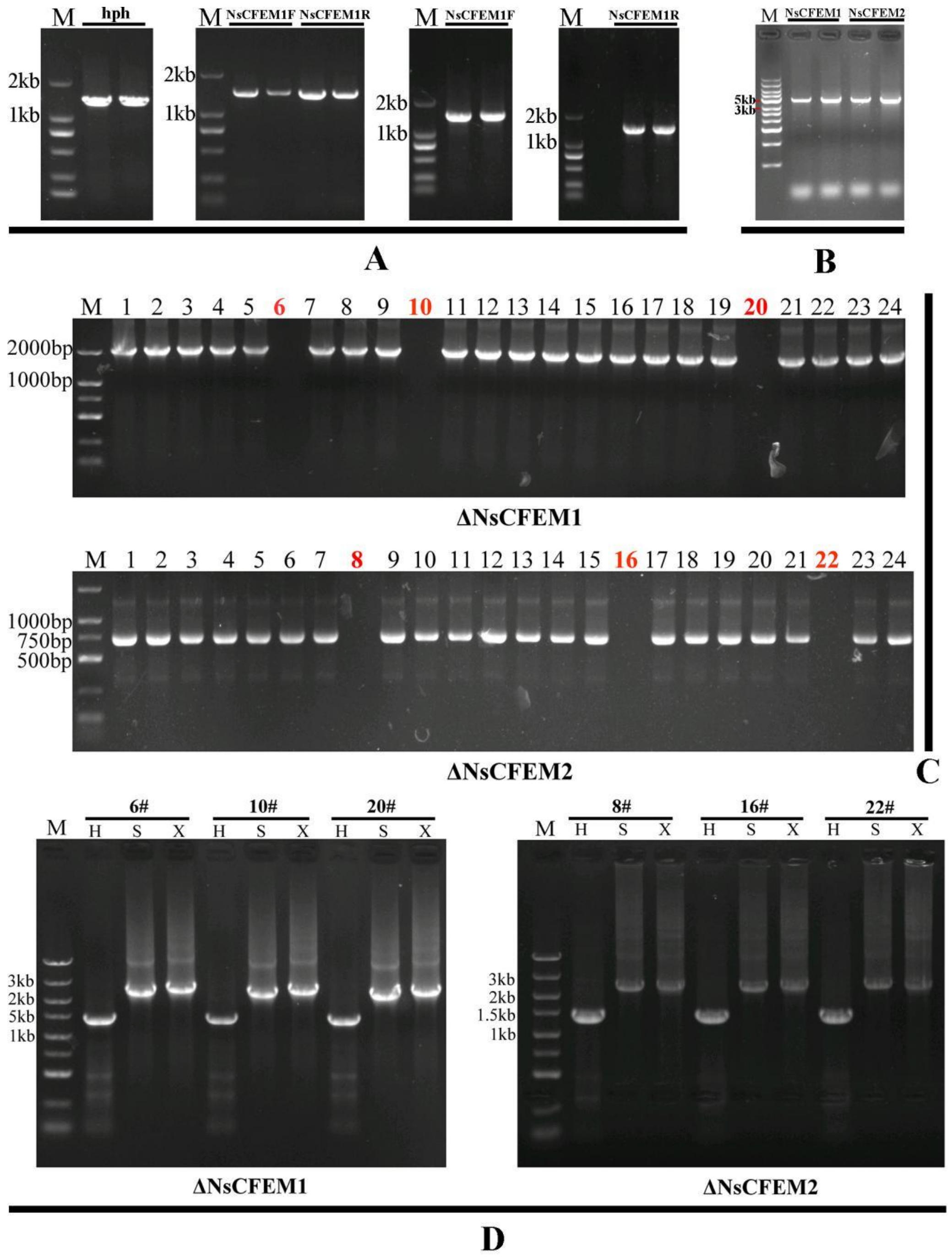

Supplementary Figure 1 Construction of knockout vector and RCR validation of knockout transformants

Note: A: Electrophoretic detection of homologous arms of target genes and *hph* fragments; B: Electrophoretic detection of fusion fragments; C: Electropherogram of the first round of PCR assay for NsCFEM1 and NsCFEM2 knockout transformants (Red numbers indicate positive knockout transformant PCR validation results); D: Electropherogram of the second round of PCR assay for NsCFEM1 and NsCFEM2 knockout transformant.

4 Supplementary Figure 3

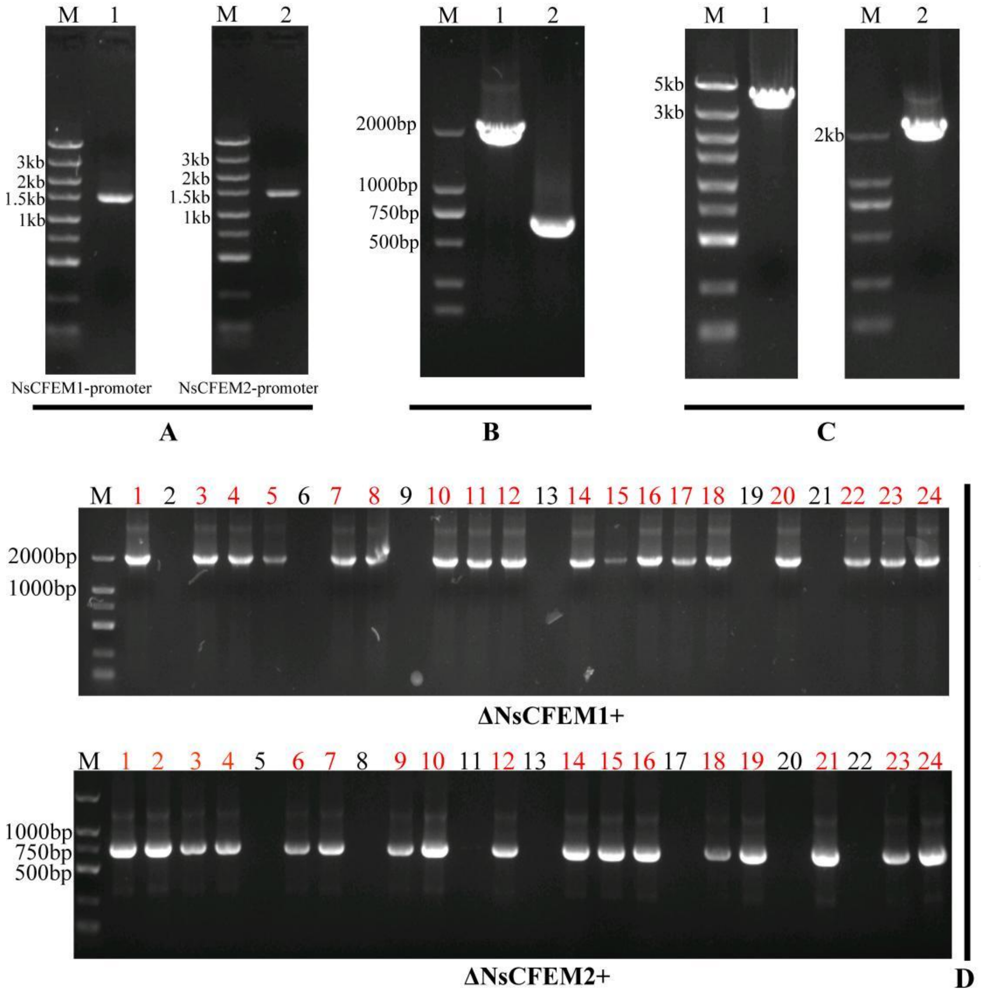

Supplementary Figure 2 Construction of complementary vector and RCR validation of complementary transformants

Note: A: The promoter fragments of *NsCFEM1* and *NsCFEM2*; B: The target genes fragments of *NsCFEM1* and *NsCFEM2*; C: The complementary sequence fusion fragments of *NsCFEM1* and *NsCFEM2*; D: Agarose gel electrophoresis of PCR assay of the complementary transformants. A-D: M: DL2000 DNA marker and DL5000 DNA marker; A-C: 1: *NsCFEM1*; 2: *NsCFEM2*. D: Red numbers indicate positive complementary transformant PCR validation results
